# Comparative effects of a candidate modified-risk tobacco product Aerosol and cigarette smoke on human organotypic small airway cultures: a systems toxicology approach†
†Electronic supplementary information (ESI) available. See DOI: 10.1039/c7tx00152e


**DOI:** 10.1039/c7tx00152e

**Published:** 2017-09-28

**Authors:** Anita R. Iskandar, Yannick Martinez, Florian Martin, Walter K. Schlage, Patrice Leroy, Alain Sewer, Laura Ortega Torres, Shoaib Majeed, Celine Merg, Keyur Trivedi, Emmanuel Guedj, Stefan Frentzel, Carole Mathis, Nikolai V. Ivanov, Manuel C. Peitsch, Julia Hoeng

**Affiliations:** a PMI R&D , Philip Morris Products S.A. (Part of Philip Morris International group of companies) , Quai Jeanrenaud 5 , CH-2000 Neuchâtel , Switzerland . Email: julia.hoeng@pmi.com ; Fax: +41 (58)242 2811 ; Tel: +41 (58)242 2214; b Biology consultant , Max-Baermann-Str. 21 , 51429 Bergisch Gladbach , Germany

## Abstract

Using an *in vitro* human small airway epithelium model, we assessed the biological impact of an aerosol from a candidate modified-risk tobacco product, the tobacco heating system (THS) 2.2, to investigate the potential reduced risk of THS2.2 aerosol exposure compared with cigarette smoke. Following the recommendations of the Institute of Medicine and the Tobacco Product Assessment Consortium, in which modified-risk tobacco products assessment should be performed in comparison with standard conventional products, the effects of the THS2.2 aerosol exposure on the small airway cultures were compared with those of 3R4F cigarette smoke. We used a *systems toxicology* approach whereby elucidation of toxic effects is derived not only from functional assay readouts but also from omics technologies. Cytotoxicity, ciliary beating function, secretion of pro-inflammatory mediators and histological assessment represented functional assays. The omics data included transcriptomic and miRNA profiles. Exposure-induced perturbations of causal biological networks were computed from the transcriptomic data. The results showed that THS2.2 aerosol exposure at the tested doses elicited lower cytotoxicity levels and lower changes in the secreted pro-inflammatory mediators than 3R4F smoke. Although THS2.2 exposure elicited alterations in the gene expression, a higher transcriptome-induced biological impact was observed following 3R4F smoke: The effects of THS2.2 aerosol exposure, if observed, were mostly transient and diminished more rapidly after exposure than those of 3R4F smoke. The study demonstrated that the systems toxicology approach can reveal changes at the cellular level that would be otherwise not detected from functional assays, thus increasing the sensitivity to detect potential toxicity of a treatment/exposure.

## Abstract 2

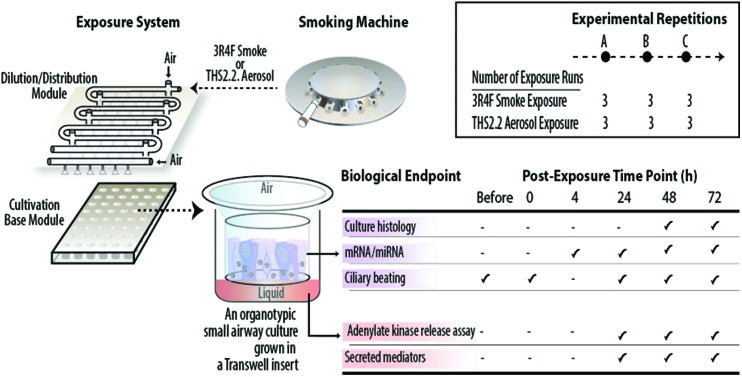
The biological impact of a tobacco heating system 2.2 aerosol and cigarette smoke were compared.

## Introduction

Studies of the human small airway region have relied on autopsy specimens or tissue samples removed by surgery.^1^ The small airway is a region of the distal lung with bronchioles smaller than 2 mm in diameter, without cartilage.^2^,^3^ Modern technologies, such as high-resolution computed tomography and helium magnetic resonance imaging, are available for studying the distal lung compartment. However, such techniques are limited to assessing the functional and morphological changes, and not suitable for a mechanistic investigation (*e.g.*, cellular and molecular assessment).^1^ Mechanistic evaluations are often conducted using animal models to assess the overall effects on the whole organism. Efforts to combine bioinformatics methods to identify the best animal models that represent specific populations of patients with respiratory diseases have been proposed.^4^ However, because of species differences, results obtained from animal studies cannot fully reflect the pathophysiology of human diseases.^5^,^6^

Alternatively, *in vitro* cellular models can overcome the inter-species differences because such models can be derived from human cells. Despite the availability and convenience of traditional two-dimensional (2D) lung culture systems, they cannot mimic the spatial organization (cell–cell interaction and cell polarity) of the human airway epithelia.^7^ Advances in tissue engineering have enabled the development of more sophisticated cellular systems, *e.g.*, three dimensional (3D) organotypic culture systems and organ-on-a-chip models, that better mimic the organization and functionality of the human tissue counterparts, than conventional 2D cellular systems.^7^,^8^ One of the benefits of the 3D organotypic airway culture systems is that they are cultured at an air–liquid interface; thus, they can be directly exposed to inhalable gases and aerosol (*e.g.*, smoke, aerosols, airborne particles, or nanoparticles). This setup eliminates the need to generate liquid smoke- or aerosol-fractions that are required for an exposure study using 2D submerged airway cells. Usually, a solution containing cigarette smoke (CS) fraction or CS extract, which does not represent whole CS, is prepared and then diluted in the culture media as the exposure. Three dimensional organotypic airway cultures can be exposed to whole CS at the apical side. This setup closely resembles the actual situation in the respiratory epithelium during smoking: CS passes across the smoker's respiratory epithelium where it comes in contact with the apical side of the cultures.

Recently, an air–liquid interface 3D organotypic small airway model became available (SmallAir™ 9). The model has a structure of a pseudostratified “tissue” layer composed of basal, ciliated, goblet, and club cells. This structure is similar to that of the human small airway epithelia. The culture is grown on a transparent membrane and is supplied with culture medium underneath.^9^ Using this newly developed organotypic model of the human small airway epithelium, we conducted the present study to compare the biological impact of an aerosol generated from a candidate modified-risk tobacco product (MRTP)^10^—the tobacco heating system (THS) 2.2^11^—with that of a smoke generated from the 3R4F reference cigarette. Readers are referred to a separate publication for more detailed information on the THS2.2 product.^11^

To assess the biological impact of the exposures, we followed a *systems toxicology* approach that involves integration of large biological data sets (*e.g.*, omics data) with conventional toxicology readouts.^12^ Different from traditional toxicology where visible adverse effects of a compound are often sought (*e.g.*, cellular injury or cell death following high-dose exposure), *systems toxicology* aims to delineate cellular molecular changes in a system under stress or perturbation. The system's responses following a subtoxic level of exposure that is more relevant to the real-life situation is investigated.^13^ This concept is also highlighted under the toxicity testing in the 21^st^ Century vision and strategy^14^ and offers a “phenotypic anchoring” at the lower doses of toxicants, which “can help to explain a toxicant's mechanism of action […] before histopathological changes were seen […]”.^15^ Under such conditions, cellular and molecular responses following exposures will bring a mechanistic understanding of the exposure-induced impacts rather than findings that merely reflect the already-visible adverse effects.^16^,^17^ Accordingly, the molecular mechanistic assessment to deduce the impact of THS2.2 aerosol exposure in the present study was performed in comparison with the benchmark 3R4F smoke-induced impact at a subtoxic concentration (approximately 0.15 mg nicotine per L 3R4F smoke). Cultures were also exposed to a higher dose of 3R4F smoke to demonstrate the progression of toxicity in the human organotypic small airway cultures following smoke exposure. We hypothesized that the cellular and molecular changes ensued following THS2.2 aerosol exposure compared with those following 3R4F smoke at subtoxic doses could provide some indication of adverse outcomes.

## Materials and methods

### Organotypic human small airway cultures

The organotypic human small airway culture model SmallAir™ was purchased from Epithelix (Plan-Les-Ouates, Geneva, Switzerland). All SmallAir™ cultures used in this study were reconstituted from primary human small airway epithelial cells of bronchiolar origin from the same donor, a healthy non-smoking 55 years-old female. Even though the use of cells from a single donor would only capture a donor-specific response, the decision was made to reduce the impact of donor-to-donor variability. The cells were grown in Transwell® inserts (with a diameter of 6.5 mm) and maintained in 12-well culture plates. The SmallAir™ cultures—fully differentiated upon arrival—were cultured at the air–liquid interface at 37 °C (5% CO_2_, 90% humidity) for 12 days in a 12-well culture plate before the experiments. The cultures were maintained in SmallAir™ culture medium (0.7 mL per well) provided by the supplier, with a medium change every 2–3 days. After exposure, the medium was not changed until it was collected for various endpoint measurements (up to 72 h post-exposure).

### Reference cigarette smoke and tobacco-heated aerosol

Mainstream CS was generated from 3R4F reference cigarettes, purchased from the University of Kentucky, Lexington, KY, USA.^21^ Mainstream test aerosol was generated from a candidate MRTP, the heat-not-burn-based technology THS2.2 (Philip Morris international R&D, Neuchâtel, Switzerland). The characteristics of THS2.2, including the specification of the product components and how the product operates, were reported in a separate publication.^11^ The yields of harmful and potentially harmful constituents (HPHCs) in the THS2.2 mainstream aerosol were described in a previous publication;^22^ the influence of tobacco blends in the concentrations of HPHCs in the THS2.2 mainstream aerosol was reported in a separate publication.^23^ In the present study, 3R4F cigarettes and THS2.2 sticks were conditioned according to ISO standard 3402.^24^

### Exposure setup

**Fig. 1:**
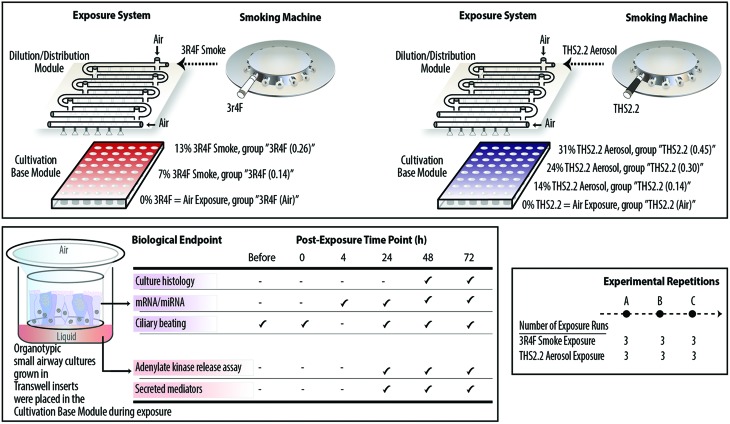
Experimental procedure and biological endpoints tested. Upper panel illustrates the smoking machines and Vitrocell® 24/48 exposure system used for exposing cultures to 3R4F smoke or THS2.2 aerosol. Lower left panel illustrates 3D organotypic cultures and the various biological endpoints assessed in the study. Lower right panel shows the number of experimental repetitions conducted for 3R4F smoke and THS2.2 aerosol exposure.

The smoke and aerosol were generated according to the Health Canada Intense smoking protocol (55 mL puff over 2 s, twice per min^25^) with an 8 s pump exhaust time. Each 3R4F cigarette was smoked to a standard butt length (approximately 35 mm), and each THS2.2 stick was aerosolized for a total of 12 puffs per stick. Two independent 30-port carousel smoking machines (SM2000; Philip Morris, International), each used for 3R4F or THS2.2, were connected to a dedicated Vitrocell® 24/48 exposure system (Vitrocell Systems GmbH, Waldkirch, Germany) (Fig. 1, upper panel). The Vitrocell® 24/48 exposure system is equipped with a Dilution/Distribution module, in which fresh air can be added into each row to dilute the smoke or aerosol. During an exposure, smoke or aerosol is passed through the Dilution/Distribution module and distributed *via* trumpets (by negative pressure) into the Cultivation Base Module,^26^ where biological culture systems were placed and exposed to smoke or aerosol at their apical sides.

This study comprised three experimental phases/repetitions (Fig. 1, lower right panel). Each experimental phase was conducted within a week. For each experimental phase, a new batch of the human small airway cultures was obtained from the supplier, and three independent exposure runs were performed (three runs for the 3R4F smoke exposure and three runs for the THS2.2 aerosol exposure, paired with their corresponding air-exposed controls) (Fig. 1, upper panel). The apical sides of the cultures were washed on the Friday preceding to the week of exposure to rinse and standardize the mucus quantity across samples. Because the exposed samples were always paired with the air-exposed controls, a plausible day-to-day variability could be minimized. This design resulted in a total of nine replicate samples for each of the endpoints and exposure conditions. The organotypic small airway cultures were exposed to smoke or aerosol for 28 min. This duration was selected according to a previous finding regarding the sensitivity of organotypic bronchial cultures following CS exposure: a 28 min exposure to 3R4F smoke induced the highest concentration of secreted matrix metalloproteinase (MMP)-1 in bronchial epithelial organotypic cultures.^27^ Various endpoints were assessed following the exposures (Fig. 1, lower left panel). Endpoints were not measured between the 4 h and 24 h post-exposure time points because sample collections and processing was practically not feasible. For gene expression (mRNA and miRNA profiling), samples were not collected immediately after exposure (0 h post-exposure) because the gene expression alterations were expected to be less robust at this post-exposure time point: a previous study reported that a low enrichment score was found in 3R4F CS-exposed samples 0.5 h post-exposure.^27^ Furthermore, a previous study showed that the CS-induced nuclear factor erythroid 2-related factor 2 (Nrf2) promoter activation in bronchial epithelial organotypic cultures at 18 h post-exposure and 24 h post-exposure were not markedly different.^28^ Nrf2 orchestrates cellular defense against cellular-damaging compounds, and is known to be upregulated upon CS exposure.^29^

A set of small airway cultures was exposed to two dilutions of 3R4F smoke and to air, simultaneously, in one exposure plate (Fig. 1, upper panel):

• A 3R4F smoke dilution (7%) corresponding to a nicotine concentration of around 0.14 mg nicotine per L, termed the “3R4F (0.14)” group,

• A 3R4F smoke dilution (13%) corresponding to a nicotine concentration of around 0.26 mg nicotine per L, termed the “3R4F (0.26)” group,

• A 0% 3R4F smoke exposure, representing the air-exposed controls for the 3R4F exposure, term the “3R4F (Air)” group.

The concentrations of 3R4F smoke were selected based on previous studies using nasal and bronchial organotypic cultures^18^–^20^ and used as the benchmark exposures to investigate the impact of THS2.2 aerosol exposure on the small airway cultures. The 7% 3R4F smoke dilution, a subtoxic level of 3R4F smoke exposure, was shown previously to elicit a no-observed-adverse-effect-level where sufficient cellular and molecular alterations were detected without culture damage.^18^–^20^ The 13% 3R4F smoke dilution, a toxic level of 3R4F smoke exposure, was previously shown to induce overt effects, *i.e.*, culture damage.^18^–^20^ A pilot concentration range study was also performed to confirm that similar findings would be observed in small airway cultures (ESI Fig. 1†).

The nicotine yield from one THS2.2 stick is approximately 30% lower than the yield from one 3R4F cigarette.^11^,^22^ Because THS2.2 is “designed to significantly reduce or eliminate the formation of HPHCs in the inhaled aerosol while preserving as much as possible the taste, sensory experience, nicotine delivery profile and ritual characteristics of cigarette”,^11^ nicotine was used as the standard compound to compare the biological impact of THS2.2 aerosol and 3R4F smoke in this study. A set of cultures was exposed to THS2.2 aerosol dilutions at nicotine concentrations matched to those of the diluted 3R4F smoke (at least for the two concentrations) and to air, simultaneously, in one exposure plate (Fig. 1, upper panel):

• A THS2.2 aerosol dilution (14%) corresponding to a nicotine concentration of around 0.14 mg nicotine per L, termed the “THS2.2 (0.14)” group,

• A THS2.2 aerosol dilution (24%) corresponding to a nicotine concentration of around 0.30 mg nicotine per L, term the “THS2.2 (0.30)” group,

• A THS2.2 aerosol dilution (31%) corresponding to a nicotine concentration of around 0.45 mg nicotine per L, term the “THS2.2 (0.45)” group,

• A 0% THS2.2 aerosol exposure, representing the air-exposed controls for the THS2.2 exposure, term the “THS2.2 (Air)” group.

### Nicotine measurement in the trapped smoke/aerosol

Previous correlation data (not shown) were used to estimate the corresponding nicotine concentrations in a given diluted 3R4F smoke or THS2.2 aerosol; the correlation between nicotine concentrations in the trapped smoke and dilutions of 3R4F smoke had been established before.^26^ For the present study, the actual concentrations of nicotine in each of the 3R4F smoke or THS2.2 aerosol dilutions were also determined within each week of the experimental phase, to ensure that the target concentrations were appropriately achieved. For this, a trapping experiment was conducted for each of the dilution conditions. Each diluted 3R4F smoke or THS2.2 aerosol was trapped in EXtrelut® 3NT columns (Merck Millipore, Billerica, MA, USA) as described previously.^26^ The EXtrelut® 3NT column was placed at the end (*i.e.*, the exhaust) of the first row of the Dilution/Distribution module of the Vitrocell® 24/48 exposure system. For this reason, the smoke or aerosol was not distributed to the trumpets—under which culture models would be located—but trapped in the EXtrelut® columns in the first row. Therefore, these trapping experiments are not feasible when the small airway cultures are exposed. Nicotine concentrations were measured from the eluted samples using gas chromatography-flame ionization detection as previously described.^26^

### Histology processing

The histological samples were obtained only from cultures harvested at the 48 h and 72 h post-exposure time points. We hypothesized that morphological alteration would occur at a later time point after exposure and after molecular changes took place, as reported in another study.^30^ After three rinses with phosphate-buffered saline (PBS), the culture was fixed for 2 h in freshly prepared 4% paraformaldehyde, and then collected from the insert for paraffin embedding using a Leica ASP300S tissue processor (Leica Biosystems Nussloch GmbH, Nussloch, Germany). Sections of 5 μm thickness were obtained using a microtome and mounted on glass slides. The slides were subsequently transferred to an automated slide stainer (Leica ST5020) for staining with hematoxylin (Merck Millipore) and eosin (Sigma-Aldrich, St Louis, MO, USA) (H&E), and alcian blue (Sigma-Aldrich) (AB). The stained slides were then covered with glass coverslips using a Leica CV5030 fully automated coverslipper. Digital microscopic images were generated using a Hamamatsu NanoZoomer 2.0 slide scanner (Hamamatsu Photonics, K.K., Hamamatsu City, Japan).

### Adenylate kinase (AK) release assay

AK activity in the basolateral medium of the cultures was measured from different cultures at various time points post-exposure using the ToxiLight™ bioassay kit (Lonza, Basel, Switzerland) according to the manufacturer's instructions. The luminescence signal was measured using a FluoStar Omega reader (BMG Labtech GmbH, Ortenberg, Germany). For each of the three experimental repetitions (*i.e.*, each culture batch), the values of the luminescence signal were normalized to the mean value of the positive and negative control samples as previously described;^19^,^31^ the formula is given in ESI Materials and methods 1.† For positive controls, triplicate samples within a batch were treated with Triton X-100 (at a 1% final concentration) and used to derive the value of 100% cytotoxicity. For negative controls, triplicate untreated samples were used. The mean values (from the total three experimental repetitions) of the normalized relative luminescence units were then reported in the figures as percentages.

### Ciliary beating functionality

Video recordings of the cultures were taken before exposure, immediately after exposure (0 h), and 24 h, 48 h, and 72 h post-exposure using a digital high-speed video camera (Sony CXD V60, Sony, Tokyo, Japan). Ciliary beating videos were not generated 4 h post-exposure because first, we hypothesized that the readouts would be similar to that taken 0 h post-exposure (as we observed in another study, ciliary beating and mucocilliary transport at 2, 4, 6, and 24 h post-exposure to a 7 min smoke exposure were largely comparable, data not shown) and second, because measurements at 4 h post-exposure time point would overlap with the sample collections and be logistically challenging. For the video recordings, the camera was connected to an inverted microscope system (Leica DMi8). Images were taken at a rate of 90 frames per second. The ciliary beating functionality was evaluated by four measures: the weighted frequency, the uniformity of the detected frequency, the active area, and the power of the detected signal (fast Fourier transformation [FFT]). Analyses were conducted on a total of 512 video frames recorded from the center of the insert surface. For each pixel, the mean of the 512 frames was subtracted, and an FFT and an approximate Bartlett's Kolmogorov–Smirnov test were performed on the pixel intensity. The weighted frequency was calculated as follows: the mean of the dominant frequency detected was weighted by its FFT power magnitude for each video if the pixel was active (*p* ≤ 0.001) and its dominant frequency was in the range of 0–20 Hz. The uniformity of the detected frequency was calculated using the mean of the Kolmogorov–Smirnov statistic; *i.e.*, the maximum difference of the normalized cumulative FFT spectrum and the uniform cumulative distribution function. The active area was defined as the proportion of pixels that showed an unadjusted Bartlett's Kolmogorov–Smirnov *p*-value ≤0.001. The strength of the ciliary beating signal was finally estimated as the sum of the FFT power spectrum in the range of 2.5–20 Hz.

### Measurement of secreted pro-inflammatory mediators

For each of the medium samples collected at the given post-exposure time point (24 h, 48 h, and 72 h post-exposure), an independent culture was used (*i.e.*, a cross-section sample, the medium was not changed for the entire duration of the post-exposure period). Based on previous observations (data not shown), the levels of secreted mediators 4 h post-exposure were low (*i.e.*, comparable to the levels of the air control with high variability); therefore, their concentrations were not measured at this time point. Multi-analyte profiling (MAP) of pro-inflammatory mediators secreted was performed using commercially available Milliplex panels (Merck Millipore) with Luminex® xMAP® technology (Luminex, Austin, TX, USA)-based analysis according to the manufacturer's instructions. Samples were analyzed on a FlexMap3D® equipped with xPONENT software v.4.2 (Luminex). The following analytes (mediators) were measured: chemokine (C–C motif) ligand (CCL) 5 and CCL-20; colony-stimulating factor (CSF) 2 and CSF-3; chemokine (C–X–C motif) ligand (CXCL) 1 (also known as GROα) and CXCL-10; epidermal growth factor (EGF); interleukin(IL)-1α, IL-1β, IL-6, and IL-8; vascular endothelial growth factor (VEGF) α; TNFα; soluble intercellular adhesion molecule (sICAM) 1; MMP-1 and MMP-9; tissue inhibitor of metalloproteinase (TIMP) 1, and thymic stromal lymphopoietin (TSLP). As positive controls (to assess the capacity of the cultures to respond to stimuli) from each of the experimental repetitions, three cultures were treated for 24 h with a combination of 10 ng mL^–1^ TNFα and 10 ng mL^–1^ IL-1β in PBS added to the basolateral medium; results are presented in ESI Table 1.† As negative controls, from each experimental repetition, three cultures were treated for 24 h with PBS in the basolateral medium.

### RNA/microRNA (miRNA) isolation and array analyses

Total RNA, including miRNA, was isolated from the small airway epithelium culture at 4, 24, 48, and 72 h post-exposure, using a previously published method.^19^,^20^,^31^,^32^ For the mRNA array, 100 ng of total RNA were reverse-transcribed to cDNA using an Affymetrix® HT 3′-IVT PLUS kit (Affymetrix, Santa Clara, CA, USA). The cDNA was labeled and amplified to complementary RNA (cRNA). The fragmented and labeled cRNA was hybridized to a GeneChip® Human Genome U133 Plus 2.0 Array (Affymetrix) in a GeneChip® Hybridization Oven 645 (Affymetrix) according to the manufacturer's instructions. Arrays were rinsed and stained on a GeneChip® Fluidics Station FS450 DX (Affymetrix) using the Affymetrix® GeneChip® Command Console® Software (AGCC software v-3.2, protocol FS450_0001). The RNA integrity number (RIN) values of the 215 samples (three experimental repetitions) were distributed between 6.8 and 10 (mean: 9.27).

For the miRNA array, a FlashTag™ Biotin HSR kit (Affymetrix) was used to label the miRNA. Two hundred nanograms of total RNA containing low molecular-weight RNA were biotinylated and hybridized to miRNA arrays version 4.0 (Affymetrix) in a GeneChip® Hybridization Oven 645 (Affymetrix) according to the manufacturer's instructions. Arrays were rinsed and stained on a GeneChip® Fluidics Station FS450 DX (Affymetrix) using the Affymetrix® GeneChip® Command Console® Software (AGCC software v-3.2, protocol FS450_0002).

Finally, the arrays were scanned using a GeneChip® Scanner 3000 7G (Affymetrix). Raw images from the scanner were saved as DAT files. The AGCC software automatically gridded the DAT file image and extracted probe cell intensities into a CEL file.

### Processing raw CEL files from the mRNA microarray

The raw CEL files were background-corrected, normalized, and summarized using frozen-robust multi-array analysis.^33^ Background correction and quantile normalization were used to generate microarray expression values from all arrays passing quality controls (QC), and were performed using the custom CDF environment HGU133Plus2_Hs_ENTREZG v16.0,^34^ as previously described in greater detail.^19^

### Analysis of mRNA data

For each experimental factor combination item, concentration, and post-exposure duration, a model to estimate the treatment effect was fitted with limma,^35^ by including the covariate exposure run as a blocking variable to account for the pairing during an exposure run (exposed *vs.* air control). The *p*-values for each computed effect were adjusted across genes using the Benjamini–Hochberg false discovery rate (FDR) method.^36^ Differentially expressed genes (DEGs) were defined as a set of genes whose FDR was <0.05. The mRNA array dataset is available in the Arrays Express repository (ID: E-MTAB-6098).

### Network perturbation amplitude (NPA) analysis of transcriptomic data

The NPA methodology was described in greater detail in a previous publication.^37^ Briefly, the methodology aims to contextualize transcriptome profiles (treated *vs.* control, or exposed *vs.* air control) by combining the alterations in gene expression into differential node values, *i.e.*, one value for each node of a causal network model.^38^ Relevant network models used for the analysis in this study are listed in ESI Table 2.† The differential node values are determined by fitting procedures inferring the values that best satisfy the directionality of the causal relationships contained in the network model (*e.g.*, positive or negative signs). NPA scores carry a confidence interval accounting for experimental variation, and the associated *p*-values are computed. In addition, companion statistics, derived to inform the specificity of the NPA score to the biology described in the network models, are reported as *O and K* if their *p*-values fall below the threshold of significance (0.05). A network is considered significantly affected by exposure if the three values (the *p*-value for experimental variation, *O, and K*) are below 0.05.^37^

A systems-wide metric for biological impact, the biological impact factor (BIF),^39^,^40^ summarizes the impacts of the exposure on the cellular system into a single (absolute) number, thus enabling a simple and high-level evaluation of the treatment effects across multiple time points. Calculating the BIF requires the collection of all applicable hierarchically structured network models (ESI Table 2†), and involves aggregating the NPA values of the individual networks.

### Processing raw CEL files from the miRNA microarray

The 215 delivered CEL files were read using the oligo package in the Bioconductor suite of microarray analysis tools for the R statistical software environment.^41^–^43^ QC of the miRNA raw data was performed as previously described,^19^ using the arrayQualityMetrics package,^44^ and resulted in the exclusion of nine CEL files. Normalized probe-level data were obtained by applying robust multi-array normalization and summarized at the probe-set level with the median polish method.^45^ Using the annotation provided by Affymetrix and the latest miRNA nomenclature according to miRBase v21,^46^ only the probe sets pertaining to human miRNA were kept in the expression matrix. Additionally, the probe sets that were not available on Affymetrix GeneChip® miRNA 3.0 arrays were not considered, to maintain compatibility with other published studies using organotypic cultures.^19^,^20^,^31^,^32^ Only the miRNA probe sets with significantly higher intensity values than their matched background probes must be considered as “detected”.^47^ A *p*-value threshold of 0.01 was selected to determine the detection calls based on Wilcoxon tests. If a miRNA probe set was detected in more than 50% of the samples in at least one sample group, it was kept for further analysis; otherwise, it was discarded. This process leads to a final expression matrix containing 206 columns and 594 rows, corresponding to the accepted samples and filtered miRNA probe sets, respectively. The miRNA array dataset is available in the Arrays Express repository (ID: E-MTAB-6004).

### Analysis of miRNA data

For each comparison (exposed *vs.* air control, at a given stimulus, concentration, and post-exposure time point), a submatrix was extracted from the global expression matrix by keeping only those samples belonging to the corresponding treatment or control groups, as well as the miRNA probe sets that were detected in more than 50% of the samples in at least one of the two sample groups. A linear model for differential expression was applied to the resulting submatrices using the moderated *t* statistic implemented in the limma package.^48^ The models included an additional variable to take into account the exposure runs. Subsequently, adjusted *p*-values were obtained following multiple testing corrections with the Benjamini–Hochberg FDR.^36^ miRNAs below the FDR threshold of 0.05 were considered differentially expressed. The miRNA expression changes are displayed as a heatmap figure, in which they are sorted according to their pattern; the clustering was done based on the “affinity propagation” algorithm implemented in the R “APCluster” package.^49^

### Statistical analysis

Statistical analysis was performed with SAS software version 9.2 (SAS Institute, Cary, NC, USA) on the following data: nicotine measurements, luminescence signals of the AK assay, ciliary beating function, and the Luminex-based measurement of secreted mediators. Mean values and standard error of the means are reported (unless otherwise specified). Comparisons of an exposed sample and its air control (*i.e.*, the paired-sample from the same exposure run) were conducted using a paired *t*-test. When applicable (*i.e.*, for comparing the relative cytotoxicity in the THS2.2-exposed samples with relative toxicity in the 3R4F-exposed samples), the comparison was conducted after subtracting the values of the corresponding air controls (*i.e.*, the paired samples). Next, the comparison was performed using a *t*-test corrected for non-equal variance (Satterthwaite correction). Numerical values from secreted mediator analysis (Luminex assay) were transformed using the natural log transformation.

## Results

### Nicotine concentrations in the diluted smoke/aerosol

The dilutions of smoke/aerosol that would match the target nicotine concentrations were estimated from previous experiments as reported before.^26^ In the present study, to ensure that the target nicotine concentrations were met according to the study design (see the Materials and methods section and Fig. 1), samples of the diluted 3R4F smoke and THS2.2 aerosol were taken throughout the study period, *i.e.*, within the three experimental repetition weeks.

Table 1 shows that the target nicotine concentrations were properly achieved from the specified applied dilutions of 3R4F smoke or THS2.2 aerosol. For example, the actual nicotine concentrations in the 7% dilution of 3R4F smoke were 0.14 mg L^–1^, 0.16 mg L^–1^, and 0.16 mg L^–1^ in the first, second, and third experimental repetitions, respectively. These values were aligned with the target nicotine concentration (*i.e.*, 0.15 mg nicotine per L). Similarly, the actual nicotine concentrations in the 13% dilution of THS2.2 aerosol were 0.11 mg L^–1^, 0.17 mg L^–1^, and 0.15 mg L^–1^ in the first, second, and third experimental repetitions, respectively. These concentrations corresponded to the target nicotine concentration of 0.14 mg L^–1^. The results confirmed that this approach allowed for a comparison of THS2.2 aerosol and 3R4F smoke at similar nicotine concentrations.

**Table 1:** Target and actual nicotine concentrations in 3R4F smoke or THS2.2 aerosol dilutions

	Experimental repetitions	Experiment 1	Experiment 2	Experiment 3
3R4F (0.15)	Target nicotine concentration (mg L^–1^)	0.15	0.15	0.15
	Applied dilution in Vitrocell® (vol/vol)	7%	7%	7%
	Actual nicotine measured in EXtrelut® (mg L^–1^)	0.14 ± 0.00 (*N* = 3)	0.16 ± 0.01 (*N* = 3)	0.16 ± 0.01 (*N* = 3)
3R4F (0.26)	Target nicotine concentration (mg L^–1^)	0.26	0.26	0.26
	Applied dilution in Vitrocell® (vol/vol)	13%	13%	13%
	Actual nicotine measured in EXtrelut® (mg L^–1^)	0.22 ± 0.01 (*N* = 3)	0.23 ± 0.01 (*N* = 3)	0.32 ± 0.02 (*N* = 3)
THS2.2 (0.14)	Target nicotine concentration (mg L^–1^)	0.14	0.14	0.14
	Applied dilution in Vitrocell® (vol/vol)	13%	13%	13%
	Actual nicotine measured in EXtrelut® (mg L^–1^)	0.11 ± 0.01 (*N* = 3)	0.17 ± 0.02 (*N* = 3)	0.15 ± 0.00 (*N* = 3)
THS2.2 (0.30)	Target nicotine concentration (mg L^–1^)	0.30	0.30	0.30
	Applied dilution in Vitrocell® (vol/vol)	24%	24%	24%
	Actual nicotine measured in EXtrelut® (mg L^–1^)	0.29 ± 0.01 (*N* = 3)	0.34 ± 0.02 (*N* = 3)	0.29 ± 0.02 (*N* = 3)
THS2.2 (0.45)	Target nicotine concentration (mg L^–1^)	0.45	0.45	0.45
	Applied dilution in Vitrocell® (vol/vol)	31%	31%	31%
	Actual nicotine measured in EXtrelut® (mg L^–1^)	0.45 ± 0.01 (*N* = 3)	0.48 ± 0.01 (*N* = 3)	0.41 ± 0.02 (*N* = 3)

### Effects on cytotoxicity and culture morphology

**Fig. 2:**
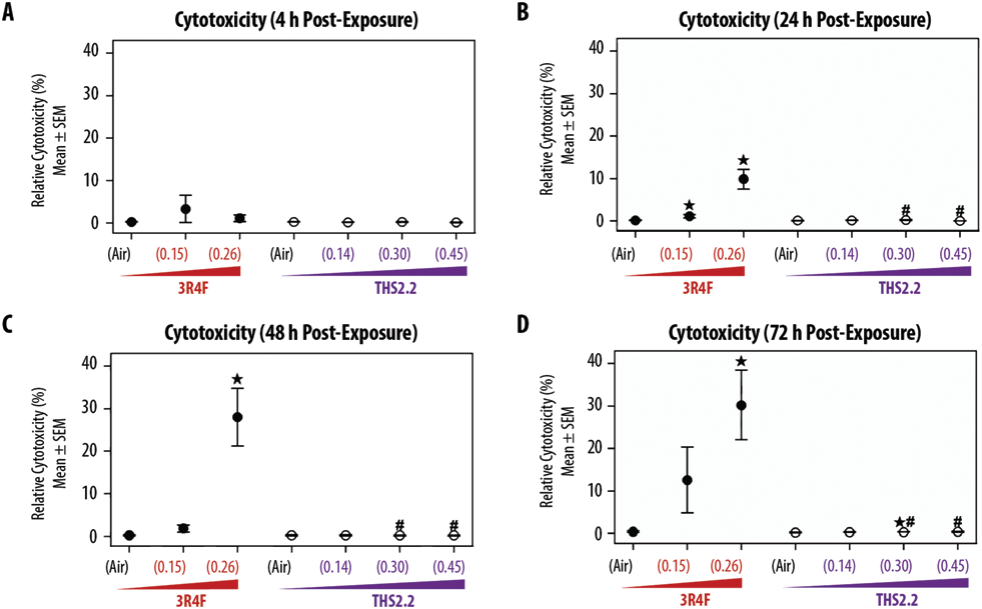
Cytotoxicity following exposure. Mean cytotoxicity levels evaluated by an adenylate kinase (AK) release assay at various time points post-exposure. AK levels were normalized relative to the positive and negative controls (see Materials and methods section). Nicotine concentrations in 3R4F smoke or THS2.2 aerosol are indicated for each group (mg L^–1^, *x*-axis). ↔ indicates *p* ≤ 0.05 compared with the corresponding air controls. # indicates a *p* ≤ 0.05 difference from 3R4F smoke exposure at a similar nicotine concentration. In the case of THS2.2 (0.45), 3R4F (0.26) was used as the comparison group.

The exposure effect was first assessed by measuring AK release as a marker of cytotoxicity and then by evaluating the morphology of the exposed small airway tissue cultures. At the earliest time point after exposure (4 h, Fig. 2A), AK release into the culture medium did not differ among the air, smoke, and aerosol exposure groups. The relative cytotoxicity levels were mainly observed in samples exposed to 3R4F (0.26) smoke; cytotoxicity increased with post-exposure duration (up until approximately 30% relative toxicity compared with the 100% cytotoxicity level in the Triton X-treated samples). Following 3R4F (0.15) smoke exposure, a noticeable increase in relative cytotoxicity was observed only 72 h post-exposure, although the difference with levels in the air controls did not reach statistically significance. THS2.2 aerosol exposure at any concentration was not associated with alterations in relative cytotoxicity at all post-exposure time points tested. One statistically significant difference was observed between the samples exposed to THS2.2 (0.30) aerosol and air (at 72 h post-exposure); however, this change was considered not biologically relevant because the relative toxicity level was only 0.34%.

**Fig. 3:**
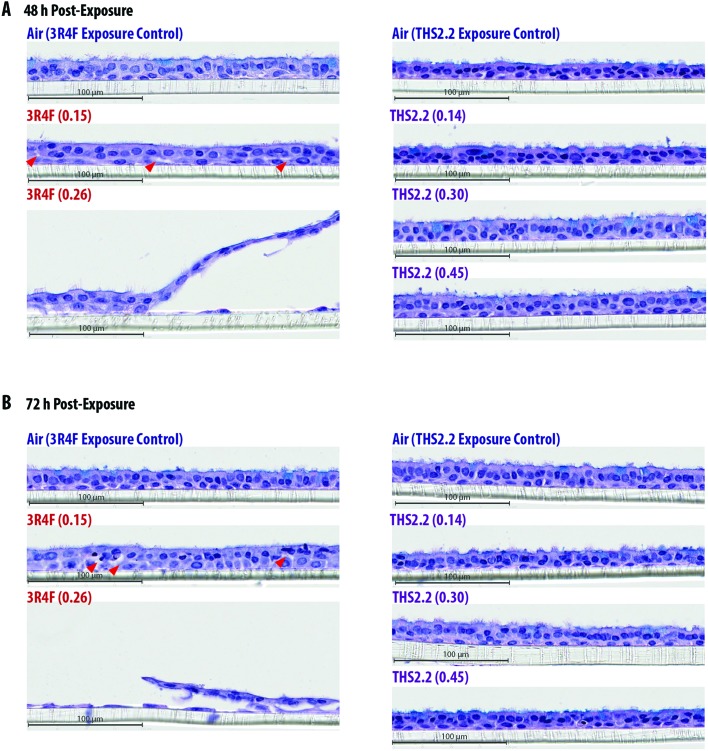
Culture morphology 48 h and 72 h post-exposure. Representative images of hematoxylin and eosin (H&E) and alcian blue (AB)-stained small airway culture sections observed 48 h (A) and 72 h (B) after exposure. Arrowheads indicate empty spaces between cells.

Histological sections from the exposed cultures were only collected at the 48 h and 72 h post-exposure time points for morphology evaluation after H&E/AB staining (Fig. 3). We assumed that alterations in culture morphology did not occur immediately after exposure; therefore, histological samples were not obtained at the earlier post-exposure time points (4 h and 24 h). The air-exposed and unexposed samples (*i.e.*, incubator controls, data not shown) exhibited similar morphology regarding the thickness of the pseudostratified epithelium, fraction of AB-positive cells, and presence of cilia. Compared with the samples exposed to air, the samples exposed to 3R4F (0.15) smoke had exhibited reduced cilia numbers and increased frequency of empty spaces between cells (see arrowheads in Fig. 3), which could be attributed to a lower cell–cell adherence, and induced detachment at the suprabasal layer. Squamous cells and apoptotic cells were also detected in the 3R4F (0.15) smoke-exposed samples. When the small airway tissue cultures were exposed to the highest concentration of 3R4F smoke (0.26 mg nicotine per L), more pronounced damage was observed 48 h (Fig. 3A) and 72 h (Fig. 3B) after exposure. In contrast, THS2.2 aerosol-exposed samples (at all concentrations tested) did not exhibit any apparent morphological alterations compared with the air-exposed controls at either post-exposure time point (Fig. 3).

### Effects on ciliary beating function

The coordinated movement (beating) of cilia serves to evacuate mucus, which traps toxicants, odorants, and other particulates from the respiratory tract.^50^ In asthmatic patients^51^ and in smokers,^52^ this defense mechanism—known as mucociliary clearance—is perturbed. Similar observations have been recorded in *in vitro* airway models exposed to whole CS or CS condensate.^20^,^53^,^54^ The small airway tissue cultures have functional cilia at their apical side, as reported by the supplier,^9^ which we also observed (Fig. 3).

**Fig. 4:**
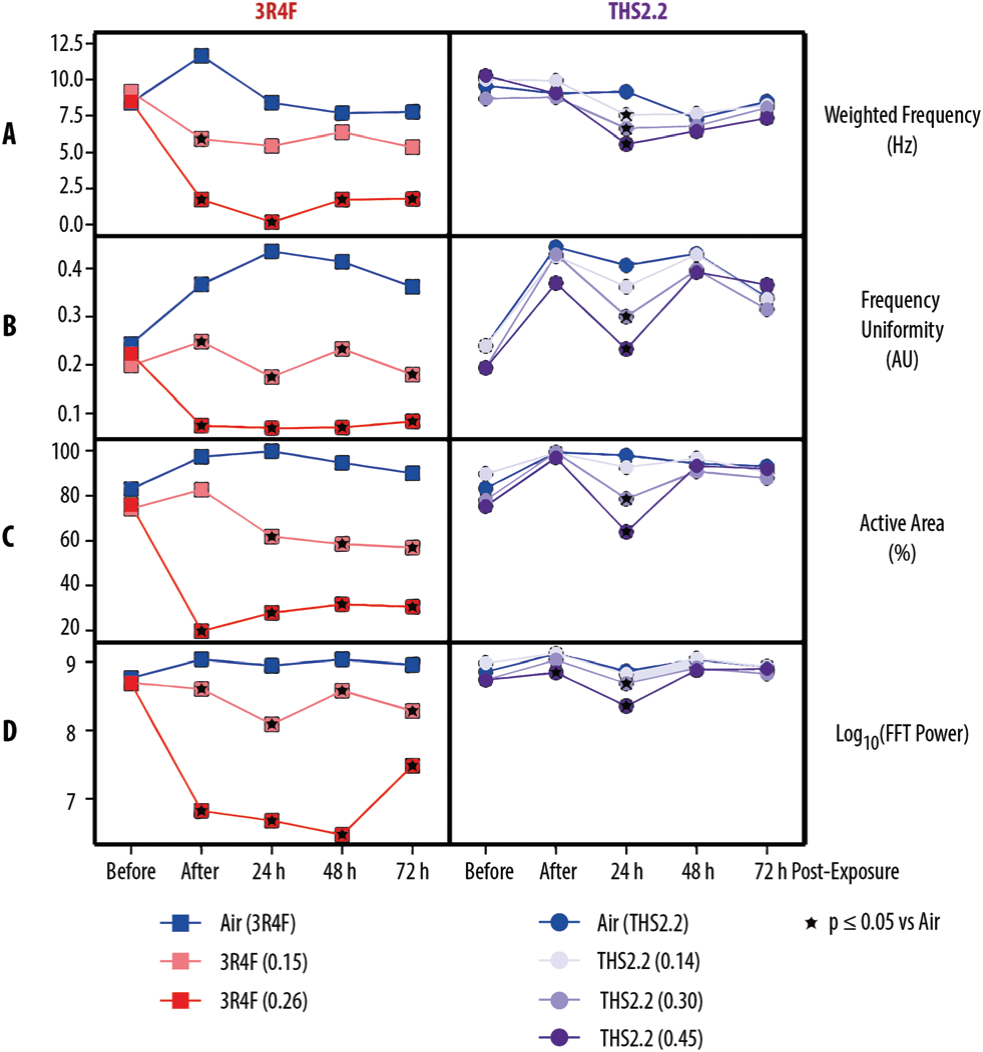
Impact of exposure on ciliary beating functionality. Ciliary beating functionality in the small airway cultures was assessed longitudinally before, immediately after (0 h), and 24 h, 48 h, and 72 h after exposure. (A) Weighted frequency (Hz) is the mean frequency over the pixel, weighted by the fast Fourier transformation (FFT) power at the pixel-dominant frequency. (B) Frequency uniformity (arbitrary unit, AU) is an index expressing the distribution of the detected frequency over the FFT spectrum (0 = blank noise; 1 = unique frequency). (C) The active area (%) is the percentage of detected pixel that differs from the blank noise. (D) The log_10_(FFT power) is an estimate of the detected power based on the beating signal of the ciliary movement. Nicotine concentrations (mg L^–1^) in 3R4F smoke or THS2.2 aerosol are indicated for each group.

Fig. 4 shows that compared with air exposure, the 3R4F smoke exposure, immediately after exposure (0 h), was linked to a reduction in the weighted frequency of ciliary beating, uniformity of the beating frequency, active area where beating was detected, and power of the beating signal in a concentration-dependent manner. These reductions (of the four different ciliary function readouts) were also observed at the 24, 48, and 72 h post-exposure time points, with the exception of a slight recovery of the power of the beating signal 72 h post-exposure (although the signal remained significantly reduced than that of the air-exposed controls).

THS2.2 aerosol exposure at all concentrations tested did not significantly affect the weighted frequency of ciliary beating, uniformity of the beating frequency, active area where beating was detected, or power of the beating signal, almost at all post-exposure time points tested (Fig. 4). An exception was seen at the 24 h post-exposure time point, where the ciliary function readouts were slightly reduced in a concentration-dependent manner compared with the readouts in the air-exposed controls.

### Inflammatory responses following exposure

**Fig. 5:**
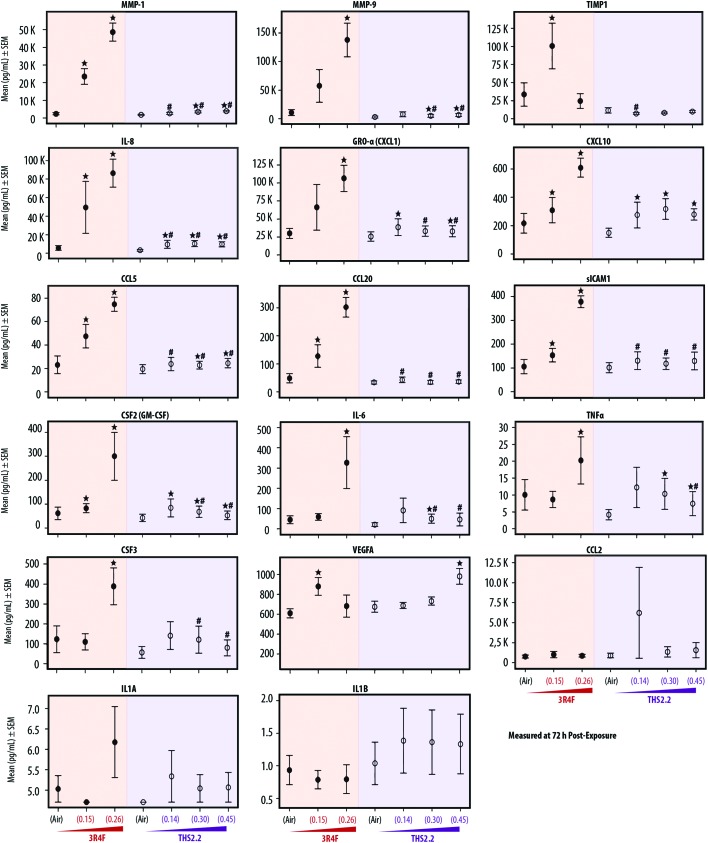
Profiles of secreted pro-inflammatory mediators following exposure. Mean concentrations of pro-inflammatory mediators measured in the basolateral media of the cultures 72 h after exposure. ↔ indicates *p* ≤ 0.05 compared with the corresponding air controls. # indicates a *p* ≤ 0.05 difference from 3R4F smoke exposure at a similar nicotine concentration. In the case of THS2.2 (0.45), 3R4F (0.26) was used as the comparison group.

Various publications have reported that organotypic airway cultures can secrete multiple cytokines, chemokines, and growth factors.^18^,^19^,^55^,^56^ In this study, we measured the concentrations of secreted pro-inflammatory mediators from the basolateral media of the cultures collected at various post-exposure time points. In general, the concentrations of the secreted mediators increased with post-exposure duration. Fig. 5 shows, the mediators measured at the 72 h post-exposure time point. The concentrations of mediators collected at the 24 h and 48 h post-exposure time points are given in ESI Table 1.† Independent of the post-exposure time points, the effects of exposure followed a similar trend: generally, we typically observed increased mediator levels following 3R4F smoke exposure, and smaller changes following THS2.2 aerosol exposure, relative to the levels seen in the air-exposed samples.

Fig. 5 shows that when compared with the mediator levels in the air-exposed controls, 3R4F smoke exposure was linked to a concentration-dependent increase in the levels of the majority of mediators measured 72 h post exposure. The concentrations of TIMP-1 and VEGFA were greater in the cultures exposed to 3R4F (0.15) smoke than in the air-exposed controls; however, their levels following 3R4F (0.26) smoke exposure did not differ from the levels following air exposure. In contrast, CSF-2, IL-6, TNFα, and CSF-3 protein levels in the media were higher in cultures exposed to 3R4F (0.26) smoke than in cultures exposed to air. THS2.2 aerosol exposure (at all concentrations tested) was linked to fewer changes in mediator levels (relative to the air exposure) for the majority of mediators, compared with the changes associated with 3R4F smoke exposure. In general, we did not observe a concentration-dependent alteration in mediator concentrations following THS2.2 aerosol exposure—except for VEGFA, for which a significant difference between THS2.2 (0.45) aerosol exposure- and the air-exposure was observed.

### Global mRNA and miRNA alterations following exposure

We aimed to deduce possible mechanisms associated with the exposure impact using a *systems toxicology* approach. We leveraged omics technologies (mRNA and miRNA microarrays) to complement the functional readouts reported in the previous sections (*i.e.*, cytotoxicity, histological assessment, ciliary beating function, and secreted pro-inflammatory mediators). As mentioned in the introduction, the mechanistic understanding of the toxicological response was evaluated following exposure at subtoxic concentrations at a no-observed-adverse-effect level, thus avoiding supraphysiological concentrations of exposures.^13^ Further, cellular and molecular alterations in severely damaged samples would depict merely the cell death response (adverse effects),^16^,^17^ which we demonstrated before.^18^ For this reason, the samples exposed to the high concentration of 3R4F smoke, *i.e.*, the 3R4F (0.26) group, were not subjected to the mRNA and miRNA microarrays because overt visible morphological changes had occurred (*i.e.*, the tissue damage shown in Fig. 2).

**Fig. 6:**
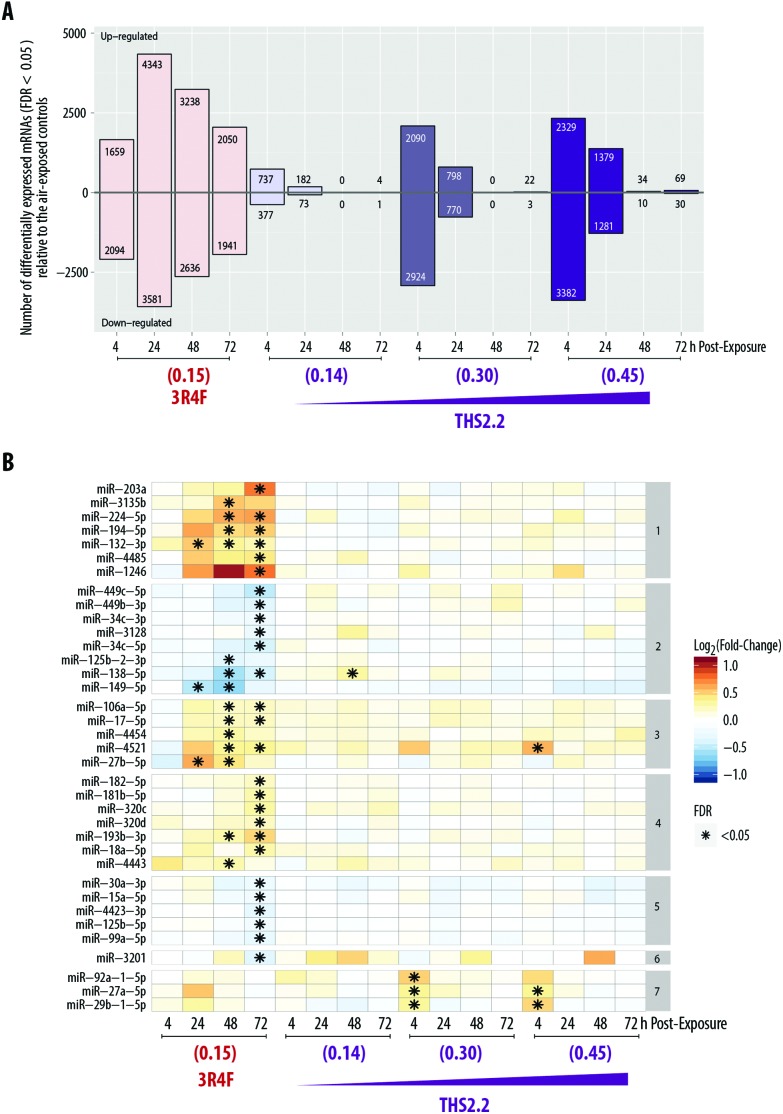
Alterations in mRNA and miRNAs expression in small airway epithelial cultures following exposure. (A) Systems response profile of the small airway cultures following exposure, referring to the number of differentially expressed genes that were up-regulated or down-regulated following exposure. (B) Significantly altered miRNAs following exposure (listed on the left side of the heatmap). The color gradient reflects the alterations in miRNA expression compared with the respective air controls. The numbering on the right side of the heatmap indicates the cluster of miRNAs in which similar patterns were observed. Nicotine concentrations in the smoke or aerosol are indicated for each group at the bottom of the heatmap (mg L^–1^). FDR, false discovery rate.

The global mRNA expression from samples exposed to 3R4F smoke or THS2.2 aerosol was compared with that of their respective air-exposed samples (*i.e.*, systems response profiles^57^). Fig. 6A shows that the greatest number of DEGs was found 24 h post-exposure to 3R4F (0.15) smoke (4343 + 3581 = 7924 genes). The number of DEGs following THS2.2 aerosol exposure at a given concentration peaked at 4 h post-exposure; these numbers also increased in a concentration-dependent manner.

In addition, miRNA profiles were generated from the same culture samples used for the mRNA analysis. Fig. 6B shows that more miRNAs were significantly altered following 3R4F (0.15) smoke exposure than following THS2.2 aerosol exposure at all concentrations tested. The largest number of miRNAs altered following 3R4F (0.15) smoke exposure was found at the 72 h post-exposure time point. Following THS2.2 aerosol exposure at any tested concentration, only a handful of miRNAs were significantly altered: miR-4521, miR-92a, miR-27a, and miR-29b (4 h post-exposure) and miR-138 (48 h post-exposure).

Our approach to quantifying systems perturbation leverages a computational methodology^37^ and a collection of causal networks depicting biological pathways/processes,^38^ described in the Materials and methods section. Using the systems response profiles (*i.e.*, the profile of gene alterations following exposure), we derived and quantified the impact of the different exposure conditions on the perturbation of causal biological networks.^37^ The degree of perturbation is termed the NPA score. Accordingly, the NPA score for a given network reflects a quantitative measure of the exposure-induced impact on the biological processes/pathways modeled in the network.

**Fig. 7:**
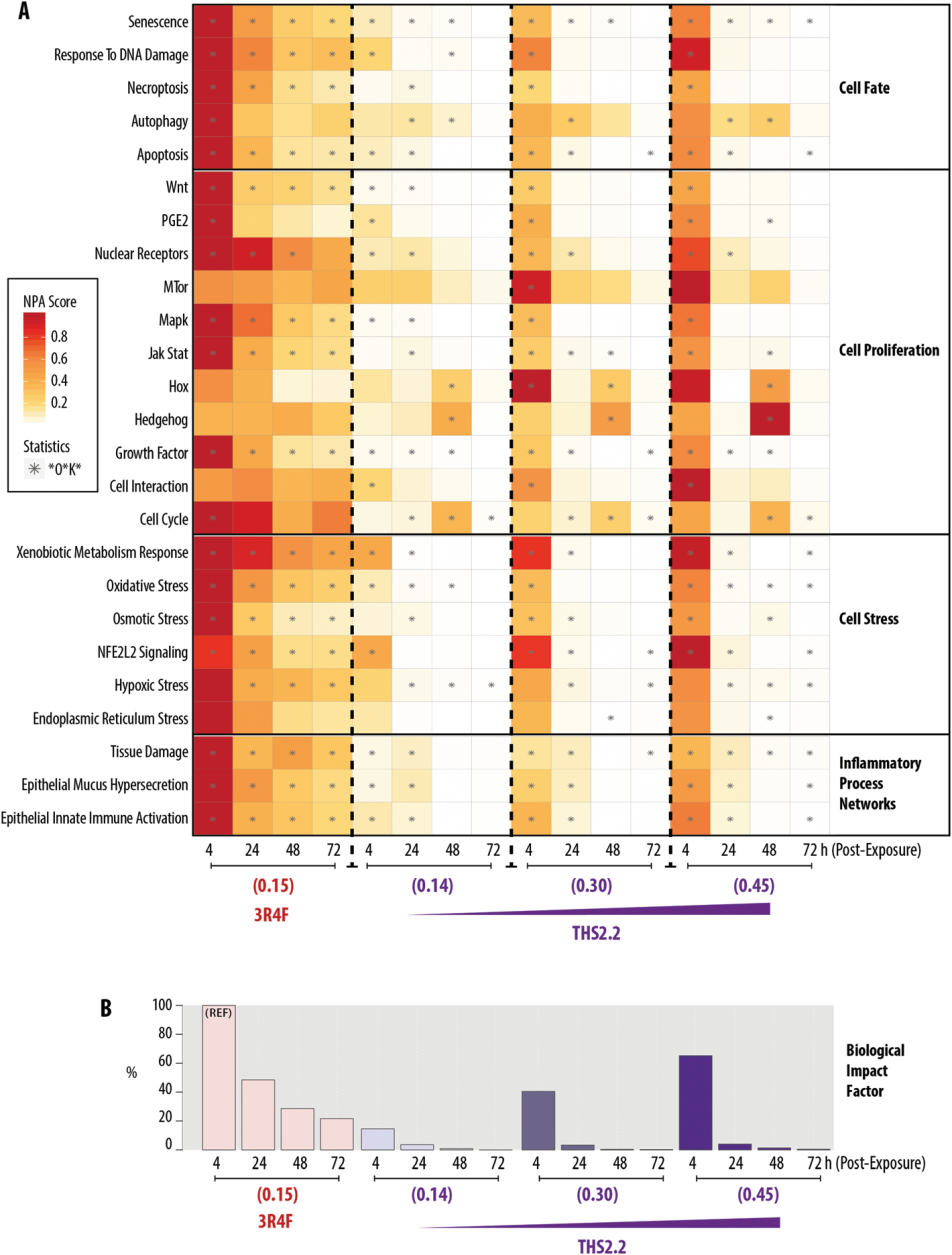
Biological Impact Factor (BIF) derived from cumulated network perturbations following 3R4F and THS2.2 exposure compared with the air controls. (A) Heatmap of network perturbation amplitude (NPA) scores of biological networks impacted by 3R4F smoke and THS2.2 aerosol exposure. The network names are listed on the left side of the heatmap, with the corresponding network family on the right side of the heatmap. The color gradient represents the NPA scores, which were normalized to the maximum NPA score per network. The star symbols (*) in the heatmap indicate that the network is significantly impacted by exposure (*i.e.*, the three values—the confidence interval, *O, and K* statistics—are below 0.05, as described in the Materials and methods section). (B) Percentage relative BIF is plotted on the *y*-axis. The highest BIF value is taken as 100% as the reference (REF). Nicotine concentrations in the smoke or aerosol are indicated for each group (mg L^–1^).

A heatmap representing the NPA scores for each exposure condition and at each post-exposure time point is shown in Fig. 7A. The network models can be grouped into four major categories: Cell Fate, Cell Proliferation, Cell Stress, and Inflammatory Process Network. Exposure to 3R4F (0.15) smoke was linked to greater NPA scores 4 h post-exposure, among the post-exposure time points tested, for the majority of networks. Exceptions were observed for mTOR, Hox, Hedgehog, Cell Interaction, and Endoplasmic Reticulum Stress networks, for which significant NPA scores were not observed following 3R4F (0.15) smoke exposure at any of the post-exposure time points tested.

At similar nicotine concentrations, THS2.2 (0.14) aerosol exposure was linked to lower NPA scores for most networks, compared with the scores observed for 3R4F (0.15) smoke exposure. For all three THS2.2 aerosol concentrations tested—THS2.2 (0.14), THS2.2 (0.30), and THS2.2 (0.45)—similar patterns of altered NPA scores were observed: almost all networks were impacted most at the 4 h post-exposure time point than at the later time points. For the Hox, Hedgehog, and Cell Cycle networks, higher perturbations were observed at 48 h post-exposure than at the other time points following THS2.2 aerosol exposure at any tested concentration. The perturbation scores for the highest concentration of THS2.2 aerosol exposure, THS2.2 (0.45), were still lower than those observed for 3R4F (0.15) smoke exposure.

A BIF, which is an agglomeration of the NPA scores of all networks, can be computed to deduce the overall impact of exposure on the systems, as described previously.^39^,^40^,^57^ Fig. 7B shows the overall BIF scores for each of the exposure conditions in the context of the biological processes/pathways represented in the networks. The highest BIF score was observed for 3R4F (0.15) smoke exposure 4 h post-exposure (thus, the BIF is considered to be 100% assigned as the reference group marked with “REF” in Fig. 7B), and declined with post-exposure duration. A similar observation was recorded for THS2.2 aerosol exposure at all concentrations tested. The highest BIF scores for THS2.2 (0.14), THS2.2 (0.30), and THS2.2 (0.45) groups were observed consistently at the 4 h post-exposure time point: 14.60%, 40.53%, and 65.19%, respectively.

## Discussion

Increasing concerns about the toxicity of airborne contaminants, such as nanoparticles, aerosols, and pollutants, have underscored the need for relevant testing approaches to assess the toxicity risk in humans. Similar importance has been placed on developing new tobacco products, including electronic cigarettes and candidate MRTPs.^10^ This study used a recently developed small airway culture model grown at the air–liquid interface (SmallAir™ 9), whereby a *systems toxicology* approach^12^ was applied. The study aimed to assess the biological impact of an aerosol from THS2.2, a candidate MRTP, compared with that of smoke from 3R4F cigarettes. The human small airway cultures were reconstituted from primary small airway epithelial cells of a single donor: a 55 years-old female non-smoker, apparently healthy. We acknowledge that the use of a single donor captures only the donor-specific response; however, data from a single-source culture would reduce the influence of a donor-to-donor variability, thus increasing the odds of differentiating the exposure effects. Predicting reliably human population responses to toxic compounds from *in vitro* data is challenging. Inherent human variations attributed to genetic and other host factors cannot be easily modeled *in vitro*, and should be addressed before a successful quantitative *in vitro*-to-*in vivo* extrapolation.^58^ Nonetheless, in our previous *in vitro* work,^20^ we determined that the exposure-related transcriptome changes were largely similar between two donors provided by two different providers, despite minor differences.

In the present study, we used a high concentration 3R4F smoke exposure to show a concentration–response relationship (of progressive toxicity until adverse effects are evident), as observed in other studies.^18^–^20^ Here we showed that marked overt effects in the cultures could be seen following exposure to 3R4F (0.26) smoke—corresponding to a 13% 3R4F smoke dilution. One indication of an adverse effect is a disruption of the system's function.^59^ This was particularly evident from the impaired ciliary beating function observed in this exposure. The levels of the beating frequency, uniformity of the frequency, active area, and power of the beating signals were lower in samples exposed to 3R4F (0.26) smoke than in those exposed to air, immediately after exposure (0 h) and 24, 48, and 72 h post-exposure. This finding suggested that mucociliary clearance following exposure to 3R4F (0.26) smoke was compromised. Mucociliary clearance is a mechanism controlled by a coordinated movement of cilia (the ciliary beating) to transport mucus along the respiratory tract.^51^ The reduced uniformity of the beating frequency likely illustrated a disturbed propulsion of the mucus layer. We further postulated that the reduced area where active beating was detected could be caused by loss of cilia; we observed pronounced damaged to the epithelial layer in this group (both 48 and 72 h post-exposure). The adverse effects following 3R4F smoke exposure at this concentration were similarly observed in our previous work.^18^ In that study, non-transient and overall perturbations of the network models were detected, confirming that molecular changes following a toxic exposure concentration (one that induces adverse effects) simply reflect the already-visible cellular injury.^16^,^17^ The non-transient perturbations further indicate that the effect is likely to be adverse.^59^

In the context of *systems toxicology* assessment, the mechanisms of action by which the exposure elicited biological impact were investigated at subtoxic concentrations (at the no observed-adverse-effect level). Such concentrations are likely to be more relevant to revealing the mechanism of the exposure-induced effects: they are sufficient to induce cellular and molecular alterations without resulting in adverse effects.^13^,^15^ Thus, in the present study, we assessed the mechanisms of the exposure-induced effects (evaluated from the global mRNA and miRNA changes) in comparison with those observed in the benchmark 3R4F (0.15) smoke-exposed samples. The network-based analysis of transcriptomes showed that 3R4F smoke exposure at 0.15 mg nicotine per L (corresponding to approximately 13% smoke dilution) had exerted the highest impact on the majority of networks (except for MTor, Hox, and Cell Interaction networks) under the conditions tested. The severity of the exposure impact (reflected from the NPA scores) decreased with post-exposure duration. This pattern of a decreasing impact was observed similarly following THS2.2 aerosol exposure (at all concentrations tested), although the NPA scores were lower and decrease more sharply than NPA scores following 3R4F (0.15) smoke exposure. Notable concentration-dependent perturbations of the Hedgehog, Hox, and Cell Cycle networks 48 h post-exposure to THS2.2 aerosol exposure were observed. The nodes in the Hedgehog network that were markedly impacted included those representing decreased expression of Gli proteins and inhibited catalytic activity of Smoothened (Smo) proteins (ESI Fig. 2†). The Hedgehog signaling pathway has been linked to repair functions in airway epithelia.^60^–^62^ We postulated that the Hedgehog network impacted following THS2.2 aerosol exposure, in conjunction with the perturbed Hox and Cell Cycle networks, simply indicates an adaptive homeostasis response.^63^ Cells can cope following exposure to multiple forms of stressors; tiny variations in oxygen, oxidants, pH, and even exercise can trigger biochemical, post-translational and gene expression changes. If the toxicity or damage is severe, irreparable injury would occur; such circumstances could trigger genetic and metabolic reactions, including the induction of DNA repair capacities.^63^ We observed that the perturbations of the response to DNA damage, necroptosis, Wnt, and Mapk networks were significant at all post-exposure time points following 3R4F (0.15) smoke exposure, but only significant at the early time points after exposure to THS2.2 aerosol. It could be further hypothesized that THS2.2 aerosol and 3R4F (0.15) smoke exposures elicited different cellular responses.

The study allowed us to reasonably explore the regulatory relationship between mRNA and miRNA changes following exposure, because the mRNA and miRNA were isolated from the same culture. The number of mRNAs altered following exposures decreased with post-exposure duration; in contrast, the number of altered miRNAs increased with post-exposure duration. This inverse relationship was more perceptible in the samples exposed to 3R4F (0.15) smoke than those exposed to THS2.2 aerosol—mainly because fewer miRNA alterations were detected in the samples exposed to THS2.2 aerosol. We hypothesized that the later changes in miRNAs were a consequence of the perturbation of the mRNA profiles observed at the earlier post-exposure time points. This delay in miRNA alteration may indicate a cellular response to resolve an earlier perturbation in the mRNA profiles.^64^ A similar observation in bronchial cultures following CS exposure was previously reported.^20^

Concentrations of the mediators secreted from the cells into the basolateral media increased with post-exposure duration. Nonetheless, mediator levels following THS2.2 aerosol exposure were only slightly changed (Fig. 5 and ESI Table 1†), with the exception of VEGFA. A concentration-dependent increase in secreted VEGFA proteins was observed in the culture media following THS2.2 aerosol exposure. VEGFA has been shown to promote proliferation of lung parenchymal cells^65^ and reduce alveolar epithelial apoptosis in the context of wound repair.^66^ These changes may be associated with the recovery phase following exposure (adaptive homeostasis response) postulated before. We also detected greater variability in the concentrations of CCL2, IL-1A, and IL-1B that hinders a meaningful biological interpretation. Nonetheless, the data suggested that the concentrations of secreted CCL2, IL-1A, and IL-1B were not influenced by the exposures.

The concentrations of nicotine in the smoke or aerosol cannot directly infer the actual nicotine dose delivered to the cell cultures because smoke/aerosol is evolving (partitioning of compounds is highly variable throughout the *in vitro* piping system and, similarly, throughout the human respiratory tract). Nonetheless, the subtoxic and toxic effects observed here were unlikely to be attributed to the nicotine by itself, but the entire gas phase and particulate phase delivered to the cultures. Moreover, particle size largely influences the deposition of aerosols.^67^,^68^ Although not reported here, separate work has been done to evaluate the particle size in 3R4F smoke and THS2.2 aerosol: Schaller and colleagues^22^ reported that the average mass median aerodynamic diameter of THS2.2 aerosol (0.7 μm, with a mean geometric standard deviation of 1.5) is similar to that of 3R4F smoke (0.8 μm, with a mean geometric standard deviation of 1.3). Furthermore, the efficiency of aerosol deposition in the Cultivation Base Module of the Vitrocell® 24/48 exposure system was investigated in a separate study.^69^ Steiner and colleagues reported a linear correlation between the expected deposited concentrations and the detected values of an aerosol with a mean aerodynamic particle sizes of 0.8 μm. In that study,^69^ the test aerosol was prepared from a mixture of glycerin and disodium fluorescein. Disodium fluorescein was added as a tracer; thus, measurements can be relatively easy, precise, and low-cost. Evaluating particle deposition in the exposure system remains an ongoing activity in our group. Such evaluation can be done using biological test systems (*e.g.*, cell cultures) or a surrogate matrix (*e.g.*, PBS, culture medium). Although cell cultures would be preferable because they reflect the characteristics of the biological test systems used in the respective toxicity studies, 3D organotypic cell culture models are expensive for large-scale production. We further recognize that the dose translatability from an *in vitro* exposure setup (*e.g.*, in this study) to *in vivo* conditions (*e.g.*, in smoker) cannot be extrapolated directly. First, the structure of exposure chambers is different from that of the human respiratory tract. Therefore, dynamic changes of aerosol (often referred to as aerosol evolution) along the flow path of an exposure chamber are different from those along the human's respiratory tract. Second, puffing topography differs among smokers, increasing the difficulty in simulating the smoke deposition along the respiratory tract. Puffing parameters used in experimental studies follow a smoking regimen defined by regulatory bodies that allows for standardization across laboratories and studies, but does not represent all human smoking behaviors.^70^ Such data from machine measurements are limited for product hazard assessment but “not intended to be nor valid as a measure of human exposure risk”.^70^ Our group has been working on computational modeling approaches to better characterize aerosol deposition and to estimate the total and regional aerosol deposition in human lungs, similar to the work of Pichelstorfer and colleagues.^71^ Nonetheless, despite these limitations, within the experimental setting used in the present study, the biological impact of THS2.2 aerosol exposure on small airway cultures could be compared with that of 3R4F smoke exposure.

## Conclusion

With new technologies, investigations at the systems level have become possible. We perceived biological processes/pathways involved in the exposures from the computational network analysis (the NPA methodology), although they were not evident from the more traditional toxicity readouts, *i.e.*, culture morphology, cytotoxicity, ciliary beating function, and secretion of pro-inflammatory mediators. The NPA methodology revealed not only a qualitative measure, but also a quantitative measure of the exposure impact in the context of the biological networks assessed here: the highest biological impact was observed 4 h post-exposure to 3R4F smoke at 0.15 mg nicotine per L (100% impact). In contrast, at the same post-exposure time point (4 h), THS2.2 aerosol at a comparable nicotine concentration (0.14 mg nicotine per L), elicited only a 15% relative biological impact. Overall, compared with the biological impact of 3R4F smoke, the results showed that the aerosol from the candidate MRTP THS2.2 elicited lower impact in all measured endpoints in the human small airway cultures. The effects of THS2.2, if observed, were mostly transient and diminished more rapidly after exposure. Despite the challenges and complexity of utilizing large data sets, this study demonstrated that the *systems toxicology* approach could provide an additional layer of toxicity-testing data at the pre-clinical stage.

## Author contributions

A.R. Iskandar, Y. Martinez, F. Martin, P. Leroy, M.C. Peitsch, and J. Hoeng contributed to the conception and/or design of the work. A.R. Iskandar, Y. Martinez, L.O. Torres, S. Majeed, C. Merg, K. Trivedi, E. Guedj, S. Frentzel, and N.V. Ivanov contributed to the data acquisition. A.R. Iskandar, Y. Martinez, F. Martin, W.K. Schlage, P. Leroy, A. Sewer, C. Mathis, and J. Hoeng contributed to the data analysis and interpretation. A.R. Iskandar, Y. Martinez, F. Martin, W.K. Schlage, C. Mathis, and J. Hoeng drafted the article. All authors critically reviewed and approved the article.

## Conflicts of interest

All authors are employees of Philip Morris International R&D, Philip Morris Products S.A. (part of Philip Morris International group of companies). W.K. Schlage is contracted and paid by Philip Morris International R&D. Philip Morris International is the sole source of funding and sponsor of this project.

## Supplementary Material

Supplementary information
