# Supplementary material for: Comparative effects of a candidate modified-risk tobacco product Aerosol and cigarette smoke on human organotypic small airway cultures: a systems toxicology approach
Source: Toxicol Res (Camb). 2017 Sep 28;6(6):930–46. doi: 10.1039/c7tx00152e (PMC6062162; doi:10.1039/c7tx00152e)
Supplement: Supplementary file 1 [file TX-006-C7TX00152E-s001.pdf]

Electronic Supplementary Material (ESI) for Toxicology Research.
This journal is © The Royal Society of Chemistry 2017

Supplementary materials

Supplementary Table 1. Mean concentration of the mediators secreted following exposure

			CSF3 (pg/mL)			CSF2 (pg/mL)			CXCL1 (pg/mL)			IL1A (pg/mL)		
Exposure Duration	PE	Group	N	M	SEM	N	M	SEM	N	M	SEM	N	M	SEM
28 min	24h	3R4F (Air)	9	40.99	15.49	9	16.54	5.18	9	9167.34	1412.57	9	4.7	0
28 min	24h	3R4F (0.15)	9	40.87	9.64	9	13.53	2.97	9	6964.57	1576.33	9	4.7	0
28 min	24h	3R4F (0.26)	9	39.57	8.36	9	19.44	4.26	9	11486.8	1823.74	9	4.7	0
28 min	24h	THS2.2 (Air)	9	47.92	15.06	9	18.56	7.03	9	14053.55	3173.2	9	4.7	0
28 min	24h	THS2.2 (0.14)	9	114.67	56.06	9	28.11	11.55	9	18185.3	4208.31	9	4.97	0.27
28 min	24h	THS2.2 (0.30)	9	96	51.2	9	30.35	13.12	9	19336.37	4965.38	9	5.35	0.65
28 min	24h	THS2.2 (0.45)	9	72.69	22.51	9	30.07	9.19	9	18600.28	4075.24	9	4.7	0
28 min	48h	3R4F (Air)	9	75.98	39.62	9	33.59	14.33	9	18572.47	4220.75	9	4.7	0
28 min	48h	3R4F (0.15)	9	60.21	14.26	9	40.66	7.72	9	20714.69	3213.61	9	4.7	0
28 min	48h	3R4F (0.26)	9	148.45	29.38	9	81.26	20.77	9	89092.8	43915.12	9	4.7	0
28 min	48h	THS2.2 (Air)	8	81.13	41.02	8	38.49	15.56	8	20164.44	4209.24	8	4.7	0
28 min	48h	THS2.2 (0.14)	9	78.04	33.22	9	32.26	9.28	9	20736.69	3041.07	9	4.7	0
28 min	48h	THS2.2 (0.30)	9	70.58	24.97	9	53.84	18.13	9	19642.07	3617	9	4.7	0
28 min	48h	THS2.2 (0.45)	9	117.82	44.51	9	67.02	24.14	9	25363.45	4786.29	9	4.7	0
28 min	72h	3R4F (Air)	9	122.99	66.86	9	61.63	26.11	9	29986.5	6915.68	9	5.03	0.33
28 min	72h	3R4F (0.15)	9	110	40.51	9	82.76	19.09	9	66246.96	31745.19	9	4.7	0
28 min	72h	3R4F (0.26)	9	388.42	92.3	9	299.95	100.29	9	106407.4	18227.47	9	6.18	0.87
28 min	72h	THS2.2 (Air)	9	57.34	29.63	9	42.41	15.52	9	25689.52	6530.32	9	4.7	0
28 min	72h	THS2.2 (0.14)	9	141.41	70.15	9	84.19	37.52	9	38694.71	11521.51	9	5.33	0.63
28 min	72h	THS2.2 (0.30)	9	121.21	67.79	9	67.74	23.53	9	33124.87	7250.36	9	5.04	0.34
28 min	72h	THS2.2 (0.45)	9	79.82	39.91	9	53.18	18.24	9	33010.46	7547.31	9	5.07	0.37
24 h	0h	PBS	3	64.59	46.52	3	27.61	22.94	3	11735.68	6196.68	3	4.7	0
24 h	0h	TNFα+ILß	3	86945.36	20499.8	3	270.04	158.41	3	444701.1	41365.23	3	5.03	0.33

			IL1B (pg/mL)			IL6 (mg/mL)			CXCL8 (pg/mL)			CXCL10 (pg/mL)		
Exposure Duration	PE	Group	N	M	SEM	N	M	SEM	N	M	SEM	N	M	SEM
28 min	24h	3R4F (Air)	9	0.88	0.22	9	9.53	4.38	9	1270.46	297.1	9	66.35	25.13
28 min	24h	3R4F (0.15)	9	0.64	0.19	9	14.69	2.26	9	5541.46	587.84	9	52.05	13.95
28 min	24h	3R4F (0.26)	9	0.72	0.16	9	18.05	5.33	9	7252.72	324.87	9	152.93	21.69
28 min	24h	THS2.2 (Air)	9	1.12	0.22	9	8.66	5.17	9	1459.7	285.08	9	155.89	59.13
28 min	24h	THS2.2 (0.14)	9	1.2	0.12	9	21.52	9.58	9	2896.5	763.77	9	143.11	43.31
28 min	24h	THS2.2 (0.30)	9	0.97	0.22	9	22.03	10.37	9	5572.23	2402.15	9	143.49	34.83
28 min	24h	THS2.2 (0.45)	9	0.64	0.07	9	19.35	6.82	9	4847.27	440.07	9	156.35	39.03
28 min	48h	3R4F (Air)	9	0.67	0.1	9	22.53	11.15	9	2683.61	968.64	9	128.15	37.73
28 min	48h	3R4F (0.15)	9	0.54	0.1	9	28.04	8.14	9	13475.24	2328.2	9	123.12	26.63
28 min	48h	3R4F (0.26)	9	0.53	0.07	9	65.22	13.29	9	37440.42	4589.4	9	427.68	37.39
28 min	48h	THS2.2 (Air)	8	0.92	0.27	8	28.69	15.2	8	2561.78	822.48	8	138.85	57.47
28 min	48h	THS2.2 (0.14)	9	0.72	0.21	9	27.98	12.83	9	2643.83	509.84	9	163.77	48.39
28 min	48h	THS2.2 (0.30)	9	0.89	0.28	9	36.44	15.09	9	4200.02	1012.57	9	173.24	28.05
28 min	48h	THS2.2 (0.45)	9	1.08	0.24	9	39.2	16.72	9	6865.62	2245.73	9	197.39	37.2
28 min	72h	3R4F (Air)	9	0.94	0.23	9	45.18	19.33	9	5650.99	2469.77	9	216.98	68.86
28 min	72h	3R4F (0.15)	9	0.79	0.14	9	59.25	16.73	9	49449.55	28066.23	9	309.52	89.76
28 min	72h	3R4F (0.26)	9	0.8	0.22	9	326.72	127.94	9	86356.28	15106.51	9	609.02	66.81
28 min	72h	THS2.2 (Air)	6	1.04	0.33	9	21.44	9.15	9	3094.96	840.02	9	150.2	31.48
28 min	72h	THS2.2 (0.14)	6	1.39	0.5	9	91.26	60.51	9	9535.25	4260.05	9	275.21	91.12
28 min	72h	THS2.2 (0.30)	6	1.36	0.49	9	50.48	22.77	9	10367.21	3177.67	9	316.97	72.73
28 min	72h	THS2.2 (0.45)	6	1.33	0.46	9	46.49	31.95	9	9637.3	2681.68	9	279.64	40.23
24 h	0h	PBS	3	1.27	0.31	3	28.37	25.75	3	1719.68	1184.14	3	91.64	71.47
24 h	0h	TNFα+ILß	3	8787.72	2513.99	3	3523.92	1720.65	3	192809.9	43877.95	3	893.5	60.3

Table continues

			CCL2 (pg/mL)			CCL20 (pg/mL)			MMP1 (pg/mL)			MMP9 (pg/mL)		
Exposure Duration	PE	Group	N	M	SEM	N	M	SEM	N	M	SEM	N	M	SEM
28 min	24h	3R4F (Air)	9	165.24	44.83	9	15.67	3.91	9	706.46	173.55	9	2255.09	884.49
28 min	24h	3R4F (0.15)	9	137.15	40.9	9	23.39	3.59	9	8255.48	580.42	9	2369.76	703.52
28 min	24h	3R4F (0.26)	9	183.75	17.24	9	15.81	3.4	9	4019.16	648.11	9	4283.9	1629.96
28 min	24h	THS2.2 (Air)	9	214.68	43.12	9	14.5	4.58	9	609.56	152.49	9	1536.35	662.27
28 min	24h	THS2.2 (0.14)	9	356.78	93.59	9	30.79	11.01	9	958.7	247.9	9	2566.49	1379.99
28 min	24h	THS2.2 (0.30)	9	363.47	94.96	9	27.89	11.89	9	997.15	189.49	9	1988.65	988.97
28 min	24h	THS2.2 (0.45)	9	340.32	78.15	9	20.28	5.21	9	1274.6	135.8	9	1785.7	732.81
28 min	48h	3R4F (Air)	9	352.96	95.59	9	38.49	11.75	9	1510.4	427.61	9	6730	3411.38
28 min	48h	3R4F (0.15)	9	268.56	63.35	9	69.26	11.49	9	14498.46	1243.09	9	12254.85	5516.61
28 min	48h	3R4F (0.26)	9	425.37	43.05	9	186.74	34.85	9	17179.89	2792.03	9	45974.52	8186.52
28 min	48h	THS2.2 (Air)	8	1147.36	537.67	8	29.69	6.31	8	1262.17	286.68	8	2807.45	1661.28
28 min	48h	THS2.2 (0.14)	9	319.22	56.61	9	26.95	3.42	9	1693.78	231.53	9	1770.42	284.45
28 min	48h	THS2.2 (0.30)	9	625.18	258.36	9	31.22	7.86	9	2319.66	364.46	9	4323.34	2150.25
28 min	48h	THS2.2 (0.45)	9	453.66	105.86	9	38.74	8.45	9	3417.62	523.84	9	5902.44	2317
28 min	72h	3R4F (Air)	9	762.57	184.03	9	48.79	16.07	9	2454.15	657.56	9	10770.05	5212.32
28 min	72h	3R4F (0.15)	9	1019.03	408.76	9	127.91	40.21	9	23517.59	4475.59	9	57417.96	28547.95
28 min	72h	3R4F (0.26)	9	868.14	168.22	9	302.3	35.35	9	48614	5133.73	9	137677.8	29315.98
28 min	72h	THS2.2 (Air)	9	909.6	279.05	9	33.7	5.86	9	1873.52	265.13	9	3091.15	1843.8
28 min	72h	THS2.2 (0.14)	9	6235.14	5689.06	9	43.08	9.96	9	2682.77	537.77	9	7839.39	4348.89
28 min	72h	THS2.2 (0.30)	9	1365.03	649.15	9	34.53	6.52	9	3452.52	562.77	9	5171.02	2624.73
28 min	72h	THS2.2 (0.45)	9	1572.5	944.46	9	36.93	6.02	9	3868.14	393.33	9	6615.6	2535.73
24 h	0h	PBS	3	458.67	341.62	3	28.95	16.86	2	1131.95	515.73	2	7153.68	6886.53
24 h	0h	TNFα+ILß	3	62775.76	22403.69	3	13399.45	1725	3	4062.1	337.4	3	5150.31	2261.32

			CCL5 (pg/mL)			SICAM1 (pg/mL)			TIMP1 (pg/mL)			TNFA (pg/mL)		
Exposure Duration	PE	Group	N	M	SEM	N	M	SEM	N	M	SEM	N	M	SEM
28 min	24h	3R4F (Air)	9	14.37	4.97	9	29.95	7.3	9	4176.2	1119.21	9	2.27	0.78
28 min	24h	3R4F (0.15)	9	6.35	1.52	9	37.15	9.58	9	9635.25	3300.51	9	1.68	0.38
28 min	24h	3R4F (0.26)	9	20.59	3.39	9	89.21	14.76	9	4368.32	1213.98	9	2.2	0.5
28 min	24h	THS2.2 (Air)	9	11.86	1.7	9	28.28	7.84	9	1847.39	362.26	9	2.9	1.23
28 min	24h	THS2.2 (0.14)	9	13.71	2.53	9	40.75	14.1	9	5127.09	2096.03	9	4.71	2.04
28 min	24h	THS2.2 (0.30)	9	15.97	1.97	9	35.59	12.07	9	6429.95	3252.33	9	4.38	1.79
28 min	24h	THS2.2 (0.45)	9	15.66	1.34	9	35.07	11.99	9	3713.49	925.87	9	4.61	1.51
28 min	48h	3R4F (Air)	9	19.9	5.79	9	60.07	20.41	9	12155.36	5169.17	9	5.34	2.71
28 min	48h	3R4F (0.15)	9	24.1	5.79	9	75.99	24.63	9	31959.35	6308.46	9	4.31	1.25
28 min	48h	3R4F (0.26)	9	48.21	3.3	9	218.72	32.98	9	18908.88	2738.79	9	8	1.98
28 min	48h	THS2.2 (Air)	8	13.84	3.36	8	68.82	18.05	8	23605.54	10832.05	8	6	3.12
28 min	48h	THS2.2 (0.14)	9	14.24	2.23	9	70.74	15.93	9	15967.98	6219.32	9	4.8	1.96
28 min	48h	THS2.2 (0.30)	9	20.06	3.19	9	79.14	20.27	9	19451.29	8504.07	9	8.27	3.36
28 min	48h	THS2.2 (0.45)	9	20.6	4.75	9	72.87	16.95	9	9757.39	2080.89	9	10.07	3.88
28 min	72h	3R4F (Air)	9	23.14	7.47	9	104.47	29.57		33596.24	16099.89	9	10.05	4.52
28 min	72h	3R4F (0.15)	9	47.55	9.96	9	152.46	28.46	9	100594.1	31591.81	9	8.68	2.43
28 min	72h	3R4F (0.26)	9	74.78	6.03	9	375.32	24.54	9	24513.36	10142.35	9	20.26	6.97
28 min	72h	THS2.2 (Air)	9	19.46	3.88	9	100.7	21.01	9	11604.4	3811.2	9	4.16	1.53
28 min	72h	THS2.2 (0.14)	9	23.77	5.63	9	129.49	36.47	9	7377.08	1189.89	9	12.23	5.95
28 min	72h	THS2.2 (0.30)	9	22.79	3.28	9	116.78	24.27	9	8099.24	1067.36	9	10.33	4.59
28 min	72h	THS2.2 (0.45)	9	24.58	4.13	9	128.53	36.98	9	9949.86	1351.75	9	7.45	3.57
24 h	0h	PBS	3	13.34	9	3	43.43	30.85	3	11562.04	9654.35	3	6.32	4.16
24 h	0h	TNFα+ILß	3	148.25	34.24	3	521.63	48.42	3	7859.29	3965.43	3	29640.62	10102.62

			VEGFA (pg/mL)		
Exposure Duration	PE	Group	N	M	SEM
28 min	24h	3R4F (Air)	9	141.01	6.44
28 min	24h	3R4F (0.15)	9	281.06	29.42
28 min	24h	3R4F (0.26)	9	115.32	17.28
28 min	24h	THS2.2 (Air)	9	391.46	111.65
28 min	24h	THS2.2 (0.14)	9	334.45	80.11
28 min	24h	THS2.2 (0.30)	9	425.25	103.67
28 min	24h	THS2.2 (0.45)	9	559.82	143.82
28 min	48h	3R4F (Air)	9	305.47	22.98
28 min	48h	3R4F (0.15)	9	519.86	44.11
28 min	48h	3R4F (0.26)	9	257.27	30.79
28 min	48h	THS2.2 (Air)	8	370.05	30.09
28 min	48h	THS2.2 (0.14)	9	361.09	22.51
28 min	48h	THS2.2 (0.30)	9	387.97	22.69
28 min	48h	THS2.2 (0.45)	9	479.67	22.39
28 min	72h	3R4F (Air)	9	608.62	45.25
28 min	72h	3R4F (0.15)	9	878.95	88.76
28 min	72h	3R4F (0.26)	9	680.85	111.28
28 min	72h	THS2.2 (Air)	9	674.31	55.28
28 min	72h	THS2.2 (0.14)	9	686.58	30.99
28 min	72h	THS2.2 (0.30)	9	731.45	40.19
28 min	72h	THS2.2 (0.45)	9	979.18	79.1
24 h	0h	PBS	3	113.71	15.05
24 h	0h	TNFα+ILß	3	460.99	84.29

N, sample number; M, mean; SEM, standard error of the mean. Color gradient highlights the lowest to highest mean values per given mediator measured in the basolateral media of the cultures.

Supplementary Table 2. List of network models used in the analysis

Number	Abbreviated network family name	Network name
1	CFA	Apoptosis
2	CFA	Autophagy
3	CFA	Necroptosis
4	CFA	Response To DNA Damage
5	CFA	Senescence
6	CPR	Calcium
7	CPR	Cell Cycle
8	CPR	Cell Interaction
9	CPR	Clock
10	CPR	Epigenetics
11	CPR	Growth Factor
12	CPR	Hedgehog
13	CPR	Hox
14	CPR	Jak Stat
15	CPR	Mapk
16	CPR	Mtor
17	CPR	Notch
18	CPR	Nuclear Receptors
19	CPR	PGE2
20	CPR	Wnt
21	CST	Endoplasmic Reticulum Stress
22	CST	Hypoxic Stress
23	CST	NFE2L2 Signaling
24	CST	Osmotic Stress
25	CST	Oxidative Stress
26	CST	Xenobiotic Metabolism Response
27	IPN	Epithelial Innate Immune Activation
28	IPN	Epithelial Mucus Hypersecretion
29	IPN	Tissue Damage

Abbreviations: CFA, Cell Fate; CST, Cell Stress; CPR, Cell Proliferation; IPN, Inflammatory Process Networks; Jak Stat, janus kinase/signal transducers and activators of transcription; Mapk, mitogen-activated protein kinases; Mtor, mechanistic target of rapamycin; NFE2L2, nuclear factor, erythroid 2-like 2; PGE2, prostaglandin E2. The collection of causal biological networks used here was the human network suite CBN v1.3 [38].

Supplementary Figure 1. Cytotoxicity and culture morphology obtained in the dose range-finding experiment

(A) Illustration of the exposure experiment. (B) Mean cytotoxicity levels evaluated by adenylate kinase (AK) release at various time points post-exposure. AK levels were normalized relative to the positive and negative controls (see Supplementary Materials and Methods 1). Dilutions of 3R4F smoke or THS2.2 aerosols with fresh air are indicated for each group (%, x-axis). (C) Representative images of hematoxylin and eosin (H&E) and Alcian blue (AB)-stained small airway culture sections observed 48 h after exposure.

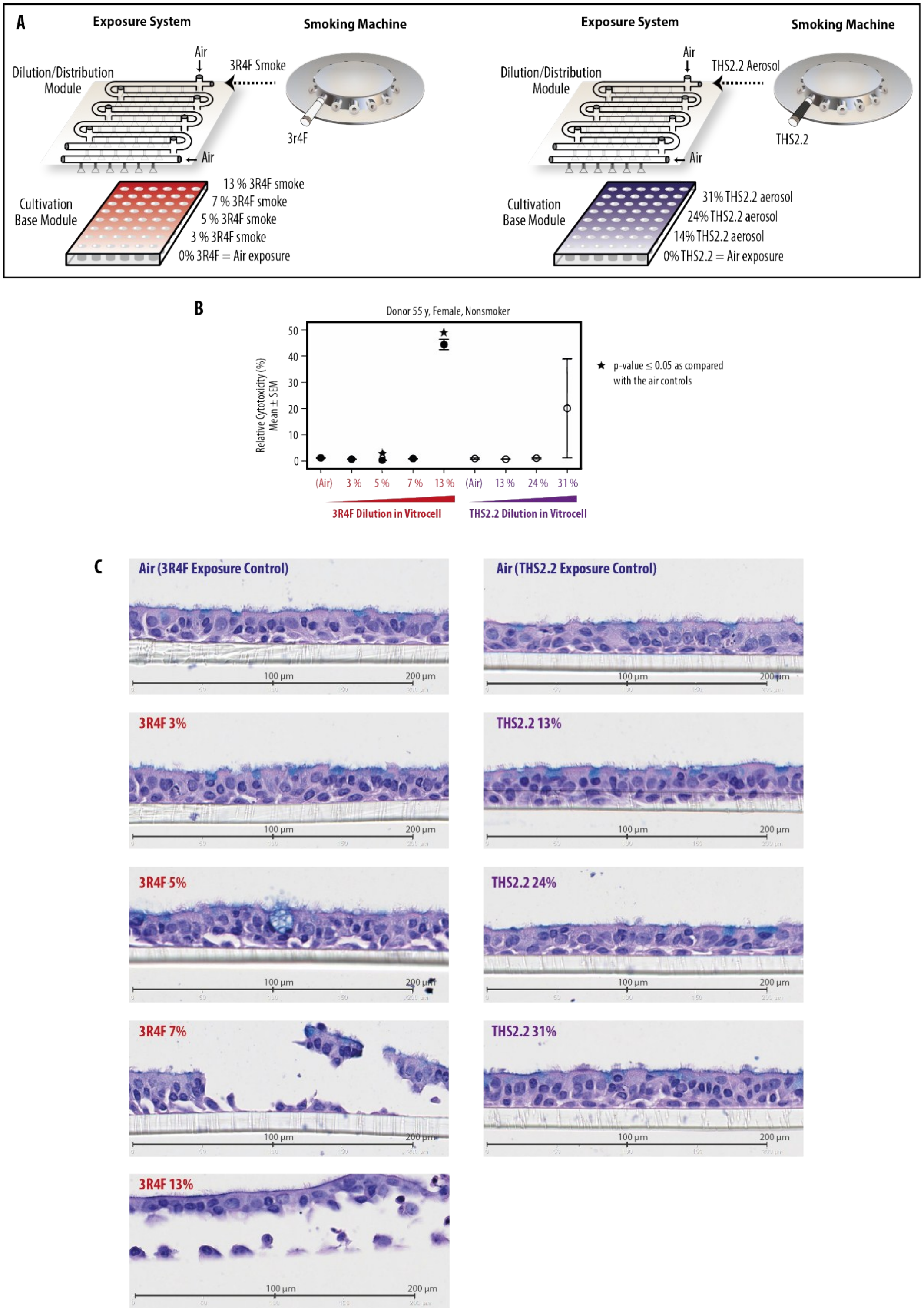
A
Exposure System
Smoking Machine
Dilution/Distribution Module
Air
3R4F Smoke
3r4F
Air
Cultivation Base Module
13 % 3R4F smoke
7 % 3R4F smoke
5 % 3R4F smoke
3 % 3R4F smoke
0% 3R4F = Air exposure
Exposure System
Smoking Machine
Dilution/Distribution Module
Air
THS2.2 Aerosol
THS2.2
Air
Cultivation Base Module
31% THS2.2 aerosol
24% THS2.2 aerosol
14% THS2.2 aerosol
0% THS2.2 = Air exposure
B
Donor 55 y, Female, Nonsmoker
Relative Cytotoxicity (%) Mean ± SEM
★ p-value ≤ 0.05 as compared with the air controls
(Air) 3 % 5 % 7 % 13 % (Air) 13 % 24 % 31 %
3R4F Dilution in Vitrocell
THS2.2 Dilution in Vitrocell
C
Air (3R4F Exposure Control)
Air (THS2.2 Exposure Control)
3R4F 3%
THS2.2 13%
3R4F 5%
THS2.2 24%
3R4F 7%
THS2.2 31%
3R4F 13%
100 µm
200 µm

Supplementary Figure 2. Perturbation of Hox, Hedgehog, and Cell Cycle networks: Comparison between the impact of 3R4F smoke and THS2.2 aerosol exposures on the small airway and bronchial cultures

(A) Heatmap of network perturbation amplitude (NPA) scores of biological networks impacted by 3R4F and THS2.2 exposure. The network names are listed on the left side of the heatmap with the corresponding network family on the right side of the heatmap. The color gradient represents the NPA scores, which were normalized to the maximum NPA score per network. The star symbols (*) in the heatmap indicate that the network is considered significantly impacted by exposure (i.e., the three values—the confidence interval, *O, and K* statistics—are below 0.05, as described in the Materials and Methods). Nicotine concentrations in the smoke or aerosol are indicated for each group (mg/L). (B) The top 10 nodes that were most impacted (blue, inhibition; red, activation) within Hox, Hedgehog, and Cell Cycle network models. The nodes were coded in BEL (biological expression language, http://openbel.org/).

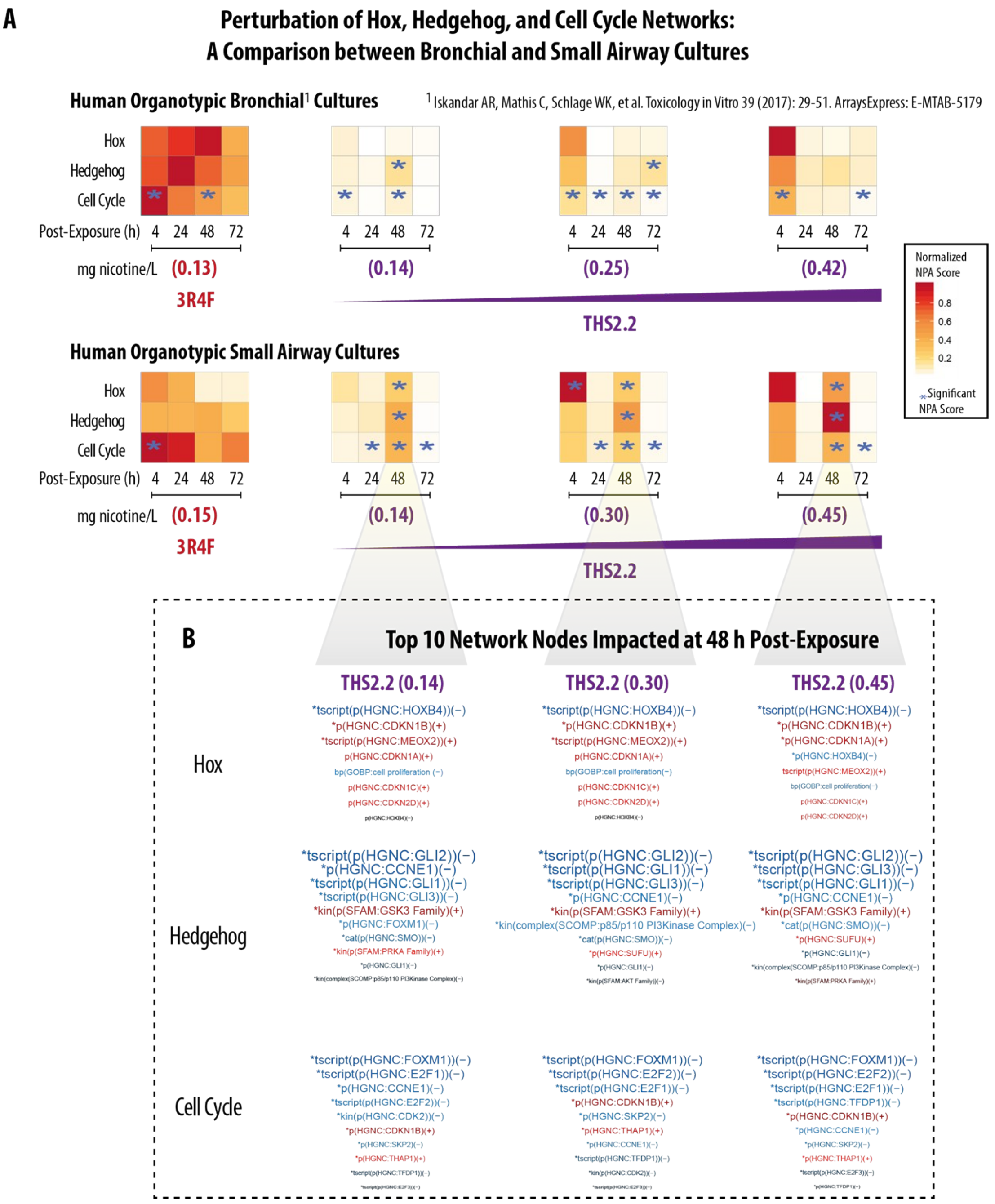
A
Perturbation of Hox, Hedgehog, and Cell Cycle Networks:
A Comparison between Bronchial and Small Airway Cultures
Human Organotypic Bronchial[1] Cultures
[1] Iskandar AR, Mathis C, Schlage WK, et al. Toxicology in Vitro 39 (2017): 29-51. ArraysExpress: E-MTAB-5179
Hox
Hedgehog
Cell Cycle
Post-Exposure (h) 4 24 48 72
mg nicotine/L (0.13) (0.14) (0.25) (0.42)
3R4F
THS2.2
Normalized NPA Score
0.8
0.6
0.4
0.2
Significant NPA Score
Human Organotypic Small Airway Cultures
Hox
Hedgehog
Cell Cycle
Post-Exposure (h) 4 24 48 72
mg nicotine/L (0.15) (0.14) (0.30) (0.45)
3R4F
THS2.2
B
Top 10 Network Nodes Impacted at 48 h Post-Exposure
THS2.2 (0.14)
THS2.2 (0.30)
THS2.2 (0.45)
Hox
*tscript(p(HGNC:HOXB4))(−)
*p(HGNC:CDKN1B)(+)
*tscript(p(HGNC:MEOX2))(+)
p(HGNC:CDKN1A)(+)
bp(GOBP:cell proliferation (−)
p(HGNC:CDKN1C)(+)
p(HGNC:CDKN2D)(+)
p(HGNC:HOXB4)(−)
*tscript(p(HGNC:HOXB4))(−)
*p(HGNC:CDKN1B)(+)
*tscript(p(HGNC:MEOX2))(+)
p(HGNC:CDKN1A)(+)
bp(GOBP:cell proliferation(−)
p(HGNC:CDKN1C)(+)
p(HGNC:CDKN2D)(+)
p(HGNC:HOXB4)(−)
*tscript(p(HGNC:HOXB4))(−)
*p(HGNC:CDKN1B)(+)
*p(HGNC:CDKN1A)(+)
*p(HGNC:HOXB4)(−)
tscript(p(HGNC:MEOX2))(+)
bp(GOBP:cell proliferation(−)
p(HGNC:CDKN1C)(+)
p(HGNC:CDKN2D)(+)
Hedgehog
*tscript(p(HGNC:GLI2))(−)
*p(HGNC:CCNE1)(−)
*tscript(p(HGNC:GLI1))(−)
*tscript(p(HGNC:GLI3))(−)
*kin(p(SFAM:GSK3 Family)(+)
*p(HGNC:FOXM1)(−)
*cat(p(HGNC:SMO))(−)
*kin(p(SFAM:PRKA Family)(+)
*p(HGNC:GLI1)(−)
*kin(complex(SCOMP:p85/p110 PI3Kinase Complex)(−)
*tscript(p(HGNC:GLI2))(−)
*tscript(p(HGNC:GLI1))(−)
*tscript(p(HGNC:GLI3))(−)
*p(HGNC:CCNE1)(−)
*kin(p(SFAM:GSK3 Family)(+)
*kin(complex(SCOMP:p85/p110 PI3Kinase Complex)(−)
*cat(p(HGNC:SMO))(−)
*p(HGNC:SUFU)(+)
*p(HGNC:GLI1)(−)
*kin(p(SFAM:AKT Family))(−)
*tscript(p(HGNC:GLI2))(−)
*tscript(p(HGNC:GLI3))(−)
*tscript(p(HGNC:GLI1))(−)
*p(HGNC:CCNE1)(−)
*kin(p(SFAM:GSK3 Family)(+)
*cat(p(HGNC:SMO))(−)
*p(HGNC:SUFU)(+)
*p(HGNC:GLI1)(−)
*kin(complex(SCOMP:p85/p110 PI3Kinase Complex)(−)
*kin(p(SFAM:PRKA Family)(+)
Cell Cycle
*tscript(p(HGNC:FOXM1))(−)
*tscript(p(HGNC:E2F1))(−)
*p(HGNC:CCNE1)(−)
*tscript(p(HGNC:E2F2))(−)
*kin(p(HGNC:CDK2))(−)
*p(HGNC:CDKN1B)(+)
*p(HGNC:SKP2)(−)
*p(HGNC:THAP1)(+)
*tscript(p(HGNC:TFDP1))(−)
*tscript(p(HGNC:E2F3))(−)
*tscript(p(HGNC:FOXM1))(−)
*tscript(p(HGNC:E2F2))(−)
*tscript(p(HGNC:E2F1))(−)
*p(HGNC:CDKN1B)(+)
*p(HGNC:SKP2)(−)
*p(HGNC:THAP1)(+)
*p(HGNC:CCNE1)(−)
*tscript(p(HGNC:TFDP1))(−)
*kin(p(HGNC:CDK2))(−)
*tscript(p(HGNC:E2F3))(−)
*tscript(p(HGNC:FOXM1))(−)
*tscript(p(HGNC:E2F2))(−)
*tscript(p(HGNC:E2F1))(−)
*tscript(p(HGNC:TFDP1))(−)
*p(HGNC:CDKN1B)(+)
*p(HGNC:CCNE1)(−)
*p(HGNC:SKP2)(−)
*p(HGNC:THAP1)(+)
*tscript(p(HGNC:E2F3))(−)
*p(HGNC:TFDP1)(−)

Supplementary Materials and Methods 1.

Normalization of the adenylate kinase (AK)-based cytotoxicity assay

For each of experimental phase (e.g., dose range finding, or each phase of the assessment studies), the value of the luminescence signal was normalized using the mean of the positive control (Triton X-100-treated cultures; considered as 100% cytotoxicity) and negative control (untreated cultures; considered as 0% cytotoxicity):

$$Cytotoxicity\ (\%) = \frac{AK_{Tissue} - AK_{Neg\ CTRL}}{AK_{Pos\ CTRL} - AK_{Neg\ CTRL}}\ x\ 100$$

, where

$$AK_{Pos\ CTRL} = \sum_{i=1}^{nbPhase} \frac{AK_{TX-100}}{nbPhase}$$

$$AK_{Neg\ CTRL} = \sum_{i=1}^{nbPhase} \frac{\sum_{j=1}^{nbCTRL^{i}} \frac{AK_{i,j}}{nbCTRL^{i}}}{nbPhase}$$

$$AK_{Tissue} = relative\ luminescence\ unit\ of\ a\ given\ sample$$

$nbPhase = number\ of\ experimental\ phase$

$Neg = negative$

$Pos = positive$

$CTRL = control$

Triton X-100 (at 1% final concentration) was added to the basolateral media of the cultures for 24 h to maximally induce cell lysis. The averages of the normalized relative luminescence unit were reported.
